# Interfacial V^5+^/V^4+^ Redox Kinetics Enabling Fe-free Photo-Fenton Catalysis in α‑V_2_O_5_‑ACNT Nanohybrids for Efficient Removal of Mixed CBZ and DFN

**DOI:** 10.1021/acsami.6c04932

**Published:** 2026-04-15

**Authors:** Deepak Kumar, Manisha Sharma, Sangeeta Shukla, Marshal Dhayal, Ajeet Kumar Kaushik, Sanjeev Kumar Sharma

**Affiliations:** † Biomaterials and Sensor Laboratory, Department of Physics, 29053 Chaudhary Charan Singh University, Meerut, Uttar Pradesh 250004, India; ‡ Laboratory of Plasma Processing and Biophysics, Department of Physics, 28817 Indian Institute of Technology (IIT) Delhi, New Delhi 110016, India; § NanoBioTech Laboratory, Department of Chemistry, 125215 Florida Polytechnic University, Lakeland, Florida 33805, United States

**Keywords:** photofenton catalysis, V_2_O_5_-ACNT hybrids, removal of diclofenac, carbamazepine antibiotics, synthesis/removal mechanism by DFT analysis

## Abstract

Efficient removal of structurally diverse and persistent pharmaceutical contaminants from wastewater, particularly in mixed-pollutant systems, remains a critical challenge in advanced water treatment technologies. Conventional Fe-based photofenton systems suffer from iron (Fe) sludge generation and secondary metal contamination, limiting their practical and sustainable application. Herein, we report Fe-free photofenton catalysis enabled by interfacial V^5+^/V^4+^ redox kinetics in α- V_2_O_5_-decorated activated carbon nanotube (α-V_2_O_5_-ACNT) nanohybrids for simultaneous removal of two emerging pharmaceutical pollutants, diclofenac (DFN) and carbamazepine (CBZ), from their mixed system. Electronic coupling at the V_2_O_5_/ACNT interface promotes rapid charge separation, abundant oxygen vacancies, and dynamic V^5+^/V^4+^ redox cycling, which efficiently activates H_2_O_2_ to generate highly reactive ^•^OH radicals without the need for Fe species. Under optimized conditions, the catalyst achieved ∼96% removal efficiency for DFN and ∼95% against CBZ within 60 min. Transient photocurrent, EPR/ESR, and radical trapping analyses confirm improved charge transport and intensified ^•^OH generation. Density functional theory calculations further reveal favorable adsorption geometries, charge redistribution, and reactive site localization of both pollutants on defect-rich surfaces, rationalizing the observed removal pathways. This study establishes mechanistic synergy between V_2_O_5_ and ACNTs as an effective strategy for designing sustainable, Fe-free photofenton systems.

## Graphical abstract

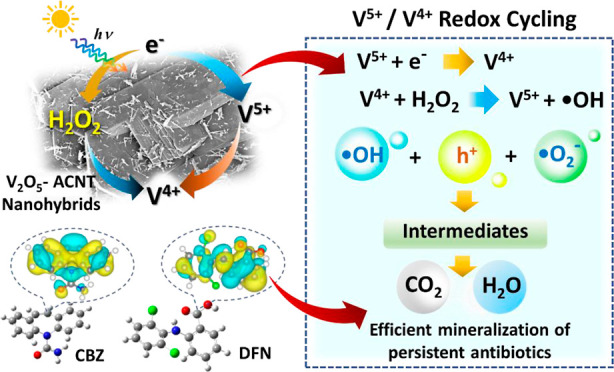

## Introduction

Widespread pharmaceutical pollutants have attracted global attention, particularly antibiotics in aquatic systems, due to their persistence, potential for bioaccumulation, and adverse ecological and human health impacts.^1–8^ Among antibiotic contaminants, nonsteroidal anti-inflammatory drugs (NSAIDs), such as diclofenac (DFN), and antiepileptic agents (e.g., carbamazepine: CBZ), are frequently detected in hospital effluents, municipal wastewater, surface and groundwater due to their widespread use and resistance to conventional treatment methods.^9–11^ These contaminants are often present as complex mixtures, making their removal from wastewater even more challenging due to different physicochemical properties and removal kinetics.^12^ Advanced oxidation processes (AOPs)^13^ have gained attention for the Fenton and photofenton reactions, which generate highly reactive hydroxyl radicals (^•^OH) that can nonselectively remove organic pollutants. However, classical Fenton systems are limited by narrow pH windows, iron (Fe) sludge formation, and low efficiency under visible light. Therefore, developing visible-light-responsive, Fe-free photofenton catalysts that are stable, reusable, and active under ambient conditions is a key research direction in environmental catalysis.^14^

Vanadium pentoxide (V_2_O_5_) is a transition-metal oxide known for its redox flexibility (V^5+^/V^4+^), visible-light absorption, and ability to activate hydrogen peroxide (H_2_O_2_) in fenton-like systems.^15,16^ However, V_2_O_5_ alone can have a low surface area and fast electron–hole recombination kinetics. Activated carbon nanotubes (ACNTs), on the other hand, provide high surface area, π-conjugated structures for strong pollutant adsorption, and excellent electrical conductivity that facilitates charge transfer.^17–20^ When combined into a V_2_O_5_-ACNT hybrid, these materials can potentially overcome individual limitations, leading to enhanced photofenton performance through synergistic effects, including improved radical generation, electron transport, and active-site accessibility. Xu et al. synthesized BiPO_4_ photocatalysts for CBZ removal via hydrothermal treatment (180 °C, 72 h), where the monoclinic phase (BPO-180–72) exhibited the highest activity, and photogenerated holes and hydroxyl radicals were identified as key reactive species.^21^

Rao et al. employed Fe (II)-activated persulfate (S_2_O_8_^2–^) for the removal of CBZ under acidic conditions (pH3), achieving high removal efficiency at an optimized molar ratio of 1:5:40 (CBZ: Fe^2+^:PS). The presence of chloride ions significantly enhanced the removal rate, whereas nitrate and phosphate ions exhibited inhibitory effects.^22^ Martínez et al. evaluated various TiO_2_-based photocatalysts under UV and near-visible light.^23^ Anatase TiO_2_ synthesized under 50% O_2_ showed superior activity with a rate constant of 0.9 min^–1^. Synergistic enhancement was observed in multiwalled carbon nanotube (MWCNT)-TiO_2_ nanocomposites. Chong et al. developed a heterogeneous fenton-like system using FeCeO*x* and H_2_O_2_, achieving 84% DFN removal at pH 5.0.^24^ The process followed pseudo-second-order kinetics during adsorption and pseudo-first-order during removal, with singlet oxygen (^1^O_2_) and superoxide radicals (^•^O_2_^–^) as the primary reactive species. Guo et al. developed a piezo-photocatalytic heterojunction composed of NH_2_–UiO-66­(Hf) and CdIn_2_S_4_, which achieved a dual functionality of H_2_O_2_ production (0.92 mmol g^–1^ h^–1^) and efficient DFN removal under visible light and ultrasonic vibration.^25^ This system leveraged interfacial In–O–Hf bonds and the intrinsic piezoelectricity of MOFs to enhance charge separation. This catalyst demonstrated high selectivity, reusability, and a mechanistic role for DFN as a hole scavenger, thereby offering a self-sustaining approach to pollutant removal and oxidant generation. Similarly, Song et al. proposed a homogeneous AOP system (DO@NaClO/DTN) for CBZ removal using sodium hypochlorite activated by dithionite under ambient conditions.^26^ Without external energy input, the system achieved high CBZ removal efficiency, utilizing naturally saturated dissolved oxygen. Mechanistic investigations confirmed that ^•^Cl and ^•^OH were the dominant reactive species, with Cl^–^ further accelerating removal. Density functional theory (DFT) calculations were also employed to support the formation of multiple reactive oxygen and chlorine species, including ^•^SO_4_^–^, ^•^O_2_^–^, and ^1^O_2_.

Although several studies have investigated the photocatalytic removal of individual pharmaceutical compounds, the mixed-contaminant systems remain underexplored, particularly in terms of the mechanistic understanding of catalyst–pollutant interactions. Furthermore, only a limited number of works have combined experimental and theoretical (DFT) approaches to elucidate pollutant adsorption behavior and electron redistribution at the catalyst interface in dual-contaminant scenarios, such as DFN and CBZ. In this research, we report the synthesis and performance evaluation of α-V_2_O_5_-decorated activated carbon nanotube (α-V_2_O_5_-ACNT) nanohybrids for the visible-light-driven Fe-free photofenton removal of a pharmaceutical mixture containing DFN and CBZ. We have comprehensively investigated the catalyst’s performance through kinetic studies, radical-scavenger tests, and electrochemical analysis, and correlated these findings with DFT simulations to gain molecular-level insight into pollutant–catalyst interactions. This study aims to establish a structure-activity-mechanism correlation to support the development of efficient, reusable, and environmentally friendly catalysts for treating complex pharmaceutical wastewater.

## Methods

### Chemical Reagents

All reagents were obtained from Sigma-Aldrich (research grade) and used directly without further purification. The reagents included ammonium metavanadate (NH_4_VO_3_) as the vanadium source, multiwalled carbon nanotubes (MWCNTs, >95% purity) as the conductive support material, and acetone (CH_3_COCH_3_, analytical grade) as the washing solvent. Sulfuric acid (H_2_SO_4_, 98%) was used for the acid treatment and surface functionalization of MWCNTs. Ethanol (≥99.9%) and deionized (DI) water were used as solvents for washing and dispersion throughout the synthesis process.

### Synthesis of Catalysts

#### Preparation of Activated Carbon Nanotubes (ACNTs)

Activated carbon nanotubes (ACNTs) were obtained by oxidative acid treatment of pristine MWCNTs (Figure 1a). In a typical procedure, 1 g of MWCNTs was dispersed in a mixed acid solution of H_2_SO_4_ and HNO_3_ (3:1 v/v) under ultrasonication for 30 min, followed by refluxing at 80 °C for 4 h. This treatment removed metallic impurities while simultaneously introducing oxygen-containing functional groups (−COOH, –OH) on the CNT surface, thereby enhancing hydrophilicity and providing anchoring sites for vanadium species. After reflux, the suspension was repeatedly washed with DI water and ethanol until the pH was neutral, and then dried at 80 °C overnight. The obtained ACNT was subsequently used to synthesize a hybrid catalyst.

**Figure 1:**
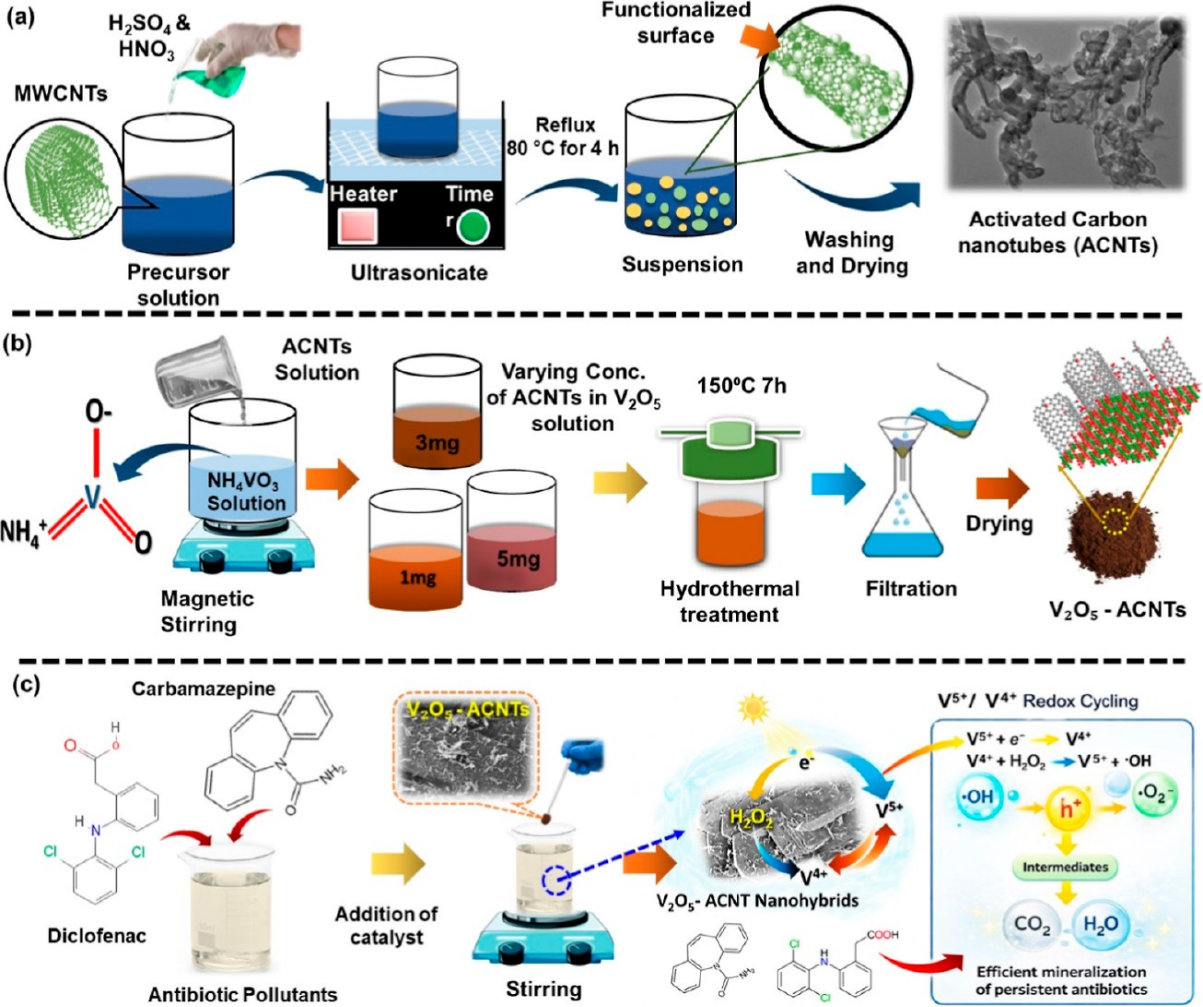
(a) Synthesis process of activated carbon nanotubes (ACNTs), (b) preparation of V_2_O_5_-ACNTs by hydrothermal method, (c) catalytic experimental process for the removal of CBZ and DFN.

#### Preparation of V_2_O_5_-ACNT Hybrids Catalysts

The V_2_O_5_-ACNT hybrids were synthesized via a modified hydrothermal method (Figure 1b). First, a 0.2 M ammonium metavanadate solution was prepared by dissolving the precursor in DI water (30 mL). Then, separate solutions of ACNTs with different loadings (1, 3, and 5 mg) were individually prepared in 10 mL DI water using ultrasonication to achieve a uniform suspension. The ACNT solution was then mixed with 30 mL of the precursor solution under constant stirring, ensuring homogeneous dispersion of the ACNTs within the precursor matrix. The resulting mixtures were transferred into Teflon-lined stainless-steel autoclaves and subjected to hydrothermal treatment at 150 °C for 7 h. After the reaction, the precipitates were collected by filtration, thoroughly washed with DI water, and dried overnight at 70 °C. Finally, calcination was performed in a muffle furnace at 400 °C for 4 h in air, to promote crystallinity and stabilize the V^5+^/V^4+^ redox-active interface between V_2_O_5_ and ACNTs. The prepared catalysts were employed to study the removal of the pharmaceutical pollutants CBZ and DFN, individually and in their mixed systems.

### Microstructural, Optical, and Chemical Bonding Analysis

Microstructural characteristics of V_2_O_5_-ACNTs nanohybrids were analyzed from XRD using Cu–Kα radiation (1.5 Å) at the scanning rate of 0.020 counts/s (Rigaku-DMAX-2400), FE-SEM (JEOL-JSM-7500) with an accelerating voltage of 14 kV along with EDAX spectra/mapping, high-resolution-transmission electron microscopy (HR-TEM) (HT7700) with a combination of selected area electron diffraction (SAED) patterns, X-ray photoelectron spectroscopy (XPS) (Thermo Scientific K-Alpha-Xps), UV–vis spectrophotometer (Igene Labserve: IG-28DS UV–vis Double Beam), Fourier-transform infrared spectroscopy (FTIR) (Labtronics LT-4100), Brunaur–Emmett–Teller (BET) (Microtra BEL Corp./BELSORPmaxII ver.1.3.4).

### Catalytic Removal Experiments

The catalytic removal activity of the V_2_O_5_-ACNTs nanohybrids was tested using a mixture of DFN (25 mg) and CBZ (25 mg) in aqueous solution (1 L) under direct sunlight irradiation, with an average sunlight intensity of ≈10^5^ lumens (Figure 1c). The catalyst dosage (5 mg) and H_2_O_2_ concentration (1 mM) were systematically optimized to maximize the removal efficiency of the target antibiotic pollutants (Figure S1), owing to a balanced interplay among active-site availability, reactive oxygen species (ROS) generation, and radical utilization efficiency. At lower catalyst dosages, insufficient active sites limit H_2_O_2_ activation,^27,28^ whereas higher dosages may lead to radical quenching, thereby reducing photofenton efficiency.^29^ At designated time intervals (10 min), samples were withdrawn, centrifuged, and analyzed via UV–vis spectroscopy. Radical-scavenging experiments were also performed using Isopropyl Alcohol (IPA) (for ^•^OH), potassium iodide (KI) (for h^+^), and Nitrogen (N_2_) (for ^•^O_2_^–^) to elucidate active species.^30^ Reusability tests and LC–MS analysis for removal intermediates were also conducted.

### DFT Calculations

DFT simulations were conducted to calculate adsorption energies, charge-density differences, and density of states (DOS) to elucidate the interaction strength, charge-transfer behavior, and electronic-structure modulation induced by molecular adsorption on the V_2_O_5_-ACNT surface. These calculations provide atomistic insights into the preferential adsorption sites and the role of V^5+^/V^4+^ redox centers in facilitating interfacial electron transfer relevant to the photofenton removal process. Electronic interactions and reactive-site preferences were analyzed to get insights into pollutant-specific removal behavior. DFT-simulated atomic structures and effective potential distributions of pristine CNT exhibit a uniform potential profile, while ACNT shows abundant surface defects and oxygen-containing functional groups, resulting in a highly nonuniform potential (Figures S2 and S3). These defect-induced electronic perturbations reduce the work function of ACNT relative to CNT, indicating an enhanced electron-donating ability.^31^ The lowered work function facilitates interfacial charge transfer from ACNT to V_2_O_5_, thereby promoting V^5+^/V^4+^ redox cycling and improving H_2_O_2_ activation during the photo-Fenton process.^32^ The atomic models of ACNTs, V_2_O_5_, and V_2_O_5_-ACNT nanohybrids were constructed to investigate their structural and electronic properties using first-principles DFT calculations. The generalized gradient approximation (GGA) with the Perdew Burke, and Ernzerhof (PBE) functional was employed to describe the exchange–correlation energy.

The optimized ACNT model (Figure 2a) consisted of a cylindrical carbon framework, with carbon atoms arranged in a hexagonal lattice and surface-functionalized with oxygen and hydrogen atoms, thereby enhancing reactivity and providing anchoring sites for V_2_O_5_ attachment. Whereas the optimized V_2_O_5_ structure (Figure 2b) exhibited an orthorhombic configuration, with vanadium atoms octahedrally coordinated to oxygen atoms. In the V_2_O_5_-ACNT nanohybrids (Figure 2c), ACNTs are uniformly dispersed over V_2_O_5_ nanoplates, forming strong interfacial interactions between the oxygen atoms of V_2_O_5_ and the carbon sites of ACNTs. A large vacuum layer was introduced along the *Z*-axis to eliminate interlayer interactions during geometry optimization. The hybrid system underwent structural relaxation without distortion, confirming the stability of the V_2_O_5_-ACNT interface.^33^ This strong coupling promotes efficient charge transfer across the interface, thereby enhancing the electronic conductivity and catalytic activity. The electronic band structures of ACNT, V_2_O_5_, and the V_2_O_5_-ACNT are shown in Figure 2d–f. The band structure of ACNTs is metallic, with several energy bands crossing the Fermi level, indicating high electrical conductivity. In contrast, the band structure of pristine V_2_O_5_ shows a well-defined bandgap of ∼2.5–3.0 eV, characteristic of a semiconducting oxide. Upon hybridization with ACNTs, the band structure of the V_2_O_5_-ACNT demonstrates a noticeable shift in energy levels and partial overlap of the conduction and valence bands near the Fermi level. This indicates enhanced electronic coupling and charge transfer between V_2_O_5_ and ACNT, thereby reducing the effective bandgap and improving the material’s electronic conductivity. Such modulation of the band structure confirms strong interfacial interactions, making the V_2_O_5_-ACNT nanohybrids promising for catalytic applications that require efficient charge transport.^34^

**Figure 2:**
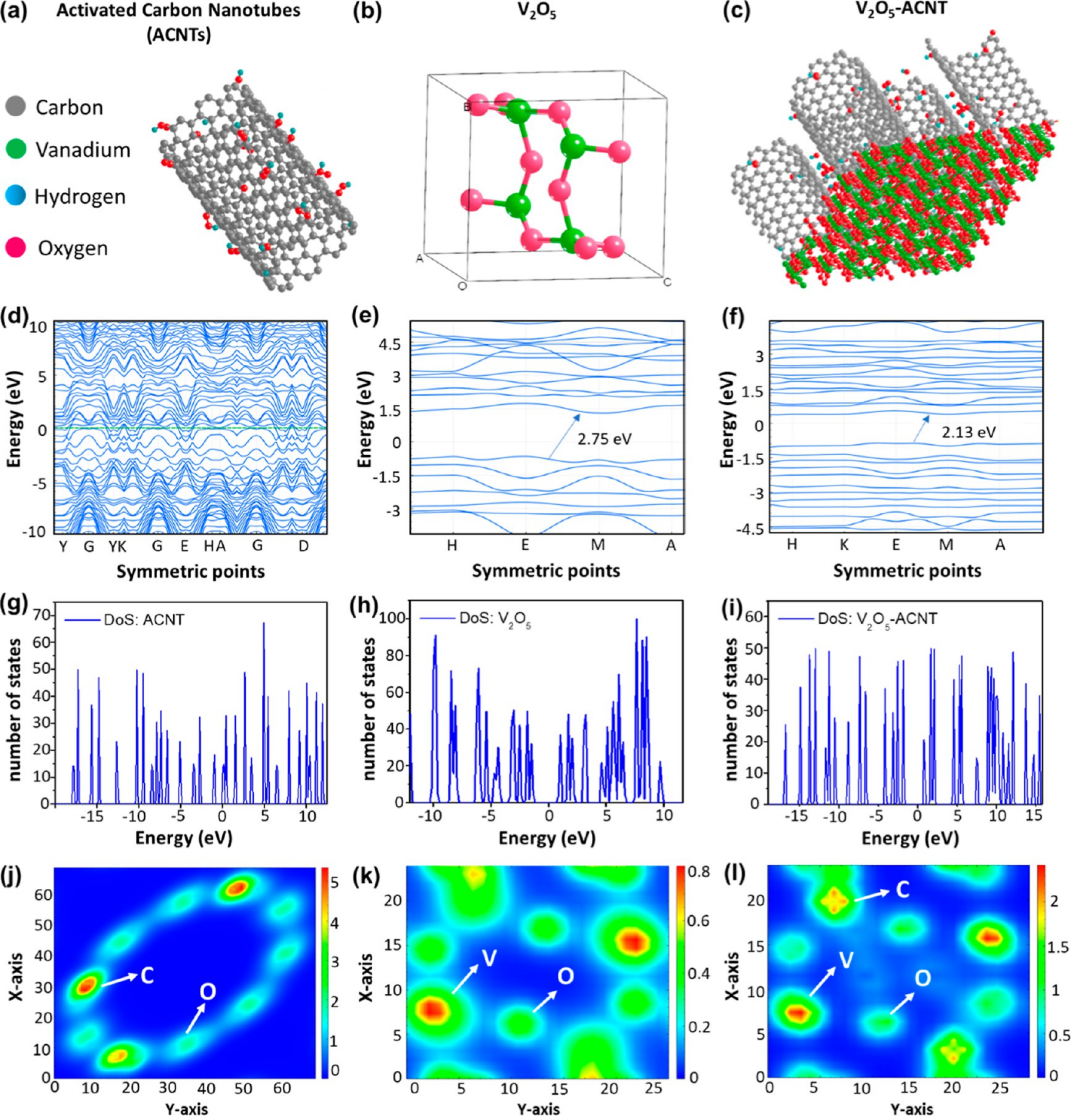
Structural, electronic, and charge distribution analyses of V_2_O_5_-ACNT nanohybrid, (a) Optimized structure of activated carbon nanotubes (ACNTs), (b) crystal structure of V_2_O_5_, (c) the interfacial configuration of the V_2_O_5_-ACNT, (d–f) Corresponding electronic band structures of ACNTs, V_2_O_5_, and V_2_O_5_-ACNT, respectively, illustrating the modulation of electronic states upon hybridization, (g–i) density of states (DOS) plots for ACNTs, V_2_O_5_, and the V_2_O_5_-ACNT, indicating enhanced charge carrier density near the Fermi level in the hybrid system, (j–l) total electron density of ACNTs, V_2_O_5_, and V_2_O_5_-ACNT, respectively, confirming efficient interfacial charge transfer and electronic coupling between V_2_O_5_ and ACNT frameworks.

The total DOS profiles for ACNT, V_2_O_5_, and the V_2_O_5_-ACNT nanohybrids are shown in Figure 2g–i. The DOS of ACNTs, exhibiting a significant number of electronic states near the Fermi level (*E*_F_ = 0 eV), confirmed the electrical conductivity. While the DOS of pristine V_2_O_5_ shows a distinct bandgap between the valence and conduction bands, indicating limited charge-carrier mobility. Upon formation of the V_2_O_5_-ACNT, the DOS profile shows a noticeable narrowing of the bandgap and the emergence of new states near the Fermi level. This shift indicates enhanced electronic coupling and interfacial charge transfer between V_2_O_5_ and ACNT, effectively improving the electronic conductivity. In addition, the total electron density distributions of ACNT, V_2_O_5_, and the V_2_O_5_-ACNT nanohybrids (Figure 2j–l) provide further insights into charge localization and interfacial interactions. For pristine ACNT, the electron density is predominantly delocalized along the conjugated carbon network, reflecting its high electronic conductivity arising from delocalized π-electrons.^35^ In contrast, pristine V_2_O_5_ exhibits localized electron density primarily around oxygen atoms, indicative of strong ionic V–O bonding and limited intrinsic charge mobility. Notably, the V_2_O_5_-ACNT shows pronounced electron density accumulation at the V_2_O_5_-ACNT interface, particularly around interfacial oxygen and carbon sites. This redistribution of charge density confirms strong electronic coupling and interfacial charge transfer between V_2_O_5_ and ACNTs. Such interfacial electron enrichment is expected to facilitate rapid electron migration, suppress charge recombination, and promote redox-active V^5+^/V^4+^ cycling.^36^

To further elucidate the activation mechanism of H_2_O_2_ on the V_2_O_5_-ACNT catalyst, DFT calculations were performed to evaluate the adsorption behavior of H_2_O_2_ on different active sites. Three possible adsorption configurations were considered (Figure 3a–c). In Configuration I, H_2_O_2_ is molecularly adsorbed at the surface V^5+^ site without dissociation. Configuration II corresponds to dissociative adsorption of H_2_O_2_ at V^4+^ sites associated with oxygen vacancies, yielding two surface-bound ^•^OH radicals. In configuration III, H_2_O_2_ weakly interacts with the ACNT surface without effective activation. The calculated adsorption energies (Figure 3d–f) reveal that configuration II exhibits the lowest adsorption energy, indicating that H_2_O_2_ activation preferentially occurs via dissociative adsorption at V^4+^/oxygen vacancy sites. This configuration facilitates efficient ^•^OH generation and promotes rapid V^5+^/V^4+^ redox cycling, which is responsible for the enhanced photofenton activity of the V_2_O_5_-ACNT nanohybrids. Notably, the superior performance of V_2_O_5_-ACNT3 also correlates with its higher lattice strain, which modulates the local electronic structure by inducing charge redistribution and stabilizing V^4+^-rich defect environments. These strain-induced electronic perturbations effectively lower the adsorption energy barrier for H_2_O_2_ dissociation, as reflected in the DFT results, thereby establishing a direct structure-electronic property-activity relationship. Accordingly, the proposed cyclic reaction pathways for configurations I–III (Figure 3g–i) highlight the variations in H_2_O_2_ activation and overall photofenton performance. Furthermore, DFT-optimized geometries of CBZ and DFN along with their successive removal intermediates and corresponding electrostatic surface potential (ESP) mappings (Figures S4 and S5) reveal the progressive redistribution of electron density and the emergence of highly positive/negative potential regions, which highlight the most susceptible reactive centers for radical (^•^OH/^•^O_2_^–^/^1^O_2_) attack, confirming selective bond cleavage at heteroatom- and aromatic-rich sites. CBZ preferentially adsorbs via its aromatic ring and amide functionality, enabling π–π interactions with the ACNT framework along with coordination near V active sites. In contrast, DFN, owing to its carboxylate group, exhibits stronger interaction with the V–O sites, resulting in more localized adsorption geometries. In the mixed system, this difference in adsorption behavior leads to competitive but nonidentical site occupation. DFN, with a relatively stronger affinity for V–O active centers, is preferentially adsorbed at these catalytic sites, while CBZ predominantly interacts with the carbonaceous ACNT surface through π–π interactions. This partial spatial separation reduces direct competition and allows simultaneous removal of both pollutants. Furthermore, the presence of oxygen vacancies (V^4+^ sites) enhances adsorption flexibility, enabling dynamic redistribution of reactants and facilitating continuous H_2_O_2_ activation and radical generation. This synergistic interaction minimizes inhibition effects typically observed in mixed systems and explains the high removal efficiency observed experimentally.

**Figure 3:**
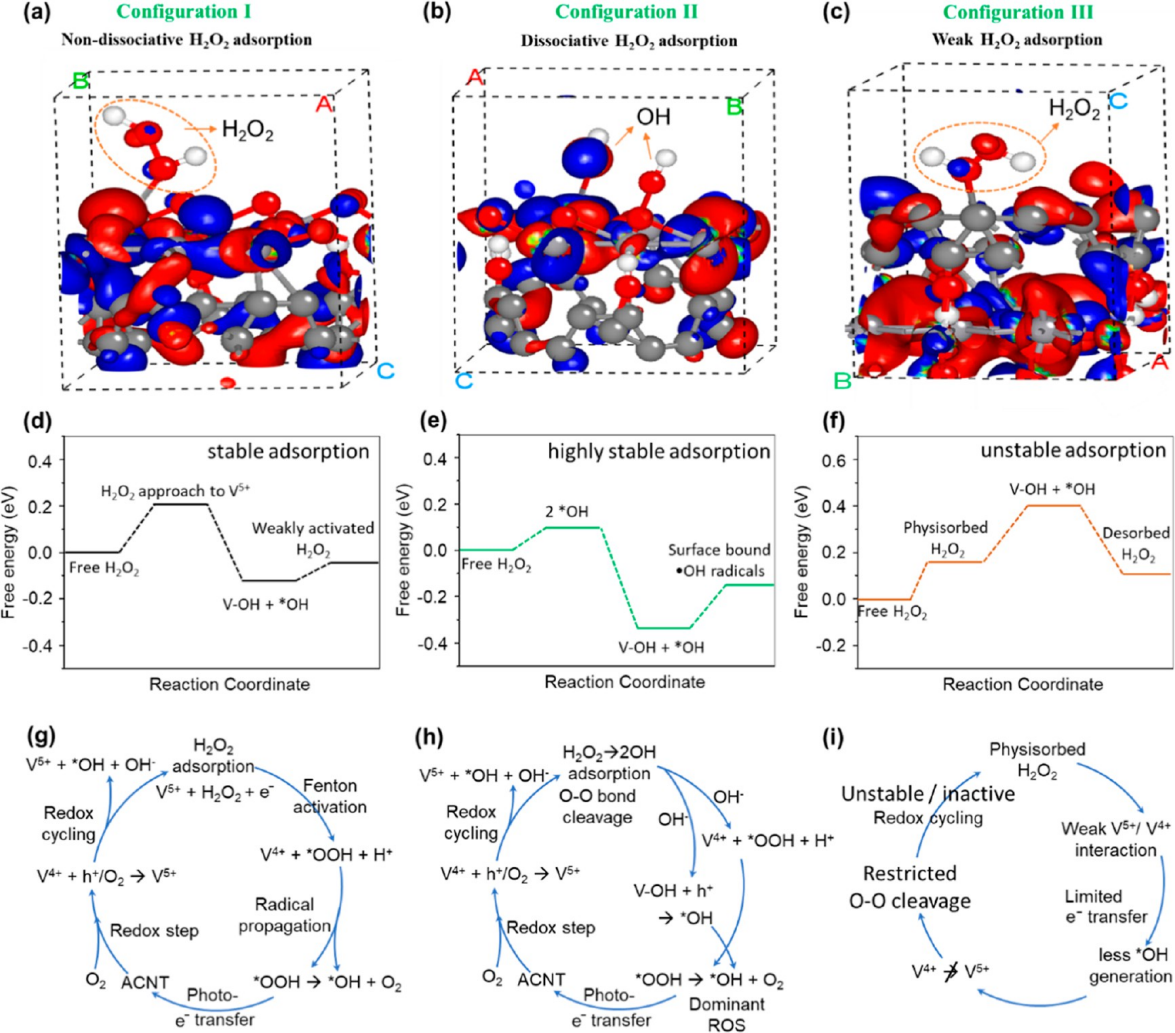
DFT-assisted H_2_O_2_ adsorption and activation on the V_2_O_5_-ACNT surface. (a–c) Three representative adsorption configurations, illustrating molecular adsorption (configuration I), dissociative adsorption with enhanced charge transfer (configuration II), and weak adsorption on the carbon-rich region (configuration III). (d–f) Corresponding Gibbs free-energy profiles, highlighting the thermodynamic preference of configuration II for H_2_O_2_ activation. (g–i) Proposed cyclic reaction pathways associated with each configuration (I–III), emphasizing the role of V^5+^/V^4+^ redox cycling in governing ^•^OH generation and photofenton activity.

## Results and Discussion

### Structural and Physicochemical Characteristics of α-V_2_O_5_-ACNT Nanohybrids

XRD patterns of pristine V_2_O_5_, ACNT, and V_2_O_5_-ACNT1, V_2_O_5_-ACNT3, and V_2_O_5_-ACNT5 nanohybrid catalysts with different ACNT loadings (1, 3, and 5 mg) are shown in Figure 4a, while Figure 4b shows an enlarged view of the highlighted region to emphasize peak evolution after ACNT loading. The characteristic diffraction peaks of orthorhombic V_2_O_5_ (JCPDS card no. 009-387) appear clearly at 2θ values corresponding to the (200), (001), (101), (110), (301), (400), and (600) planes, confirming the formation of a well-crystallized α-V_2_O_5_ phase.^37^ In the hybrid samples, both V_2_O_5_ and ACNT features are observed, indicating successful formation of V_2_O_5_-ACNT without detectable impurities or secondary phases. With increasing ACNT loading, a gradual reduction in V_2_O_5_ peak intensity and slight broadening of the V_2_O_5_ peak are observed. This behavior suggests that ACNT nanosheets are uniformly anchored to V_2_O_5_ surfaces, thereby promoting nanoscale dispersion, suppressing crystallinity, and inducing interfacial lattice distortion. Minor peak shifts further indicate the development of tensile strain within the V_2_O_5_ lattice, which may originate from oxygen vacancies and strong interfacial coupling with ACNTs. Such modifications can enhance electron mobility and active surface exposure, beneficial for catalytic processes. The retention of the orthorhombic V_2_O_5_ structure in all hybrid catalysts indicates that incorporation of ACNT does not alter the intrinsic crystal framework but improves dispersion and interfacial contact.

**Figure 4:**
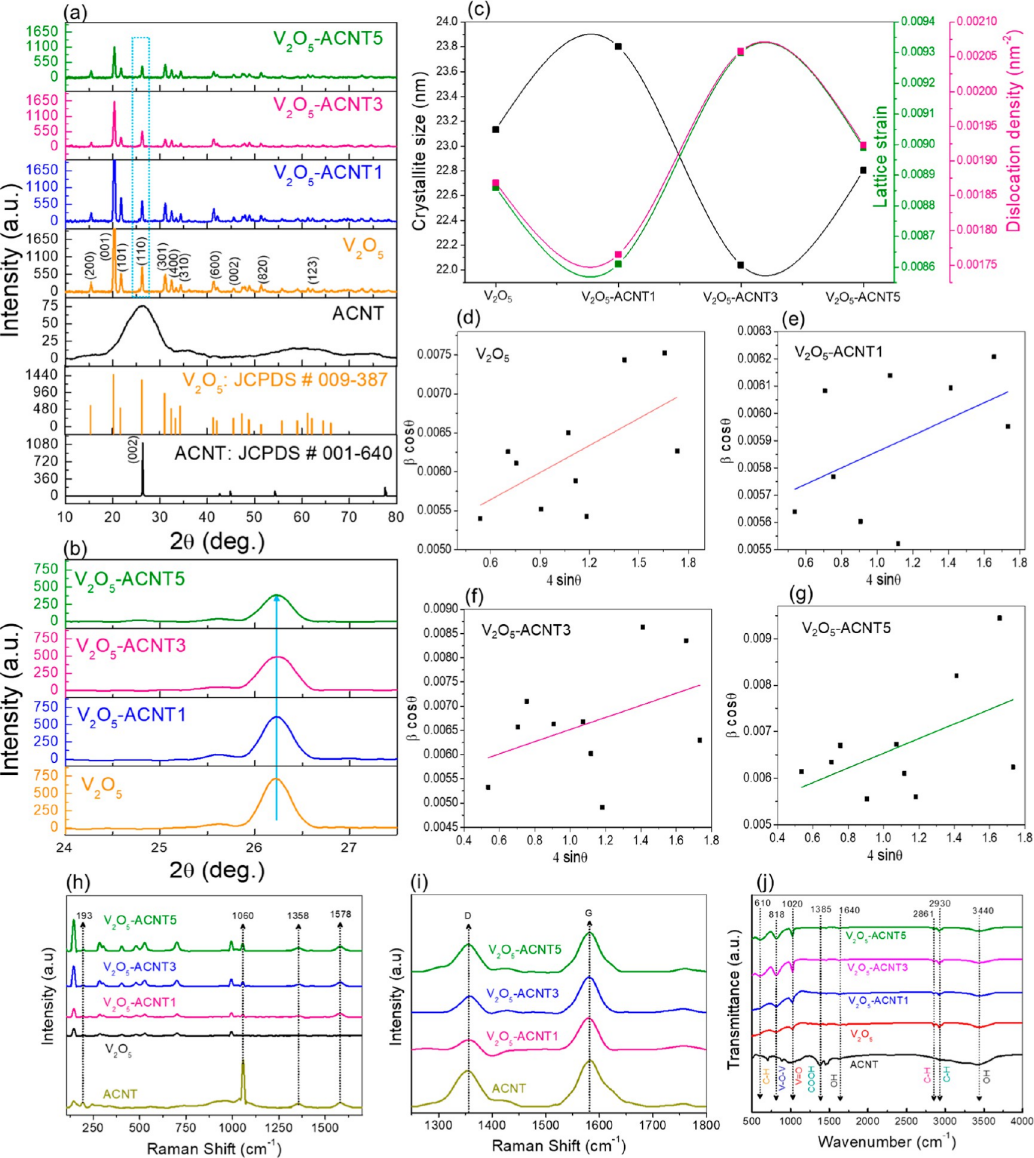
(a) XRD patterns of ACNT, V_2_O_5_, and V_2_O_5_-ACNT nanohybrids with different ACNT loadings, confirming the orthorhombic V_2_O_5_ phase (JCPDS no. 009-387) and its incorporation into the ACNT framework, (b) Enlarged view of the XRD region highlighting peak broadening with the increase in ACNT loading, (c) variation in crystallite size, lattice strain, and dislocation density as a function of ACNT wt % loading. W–H plots for (d) V_2_O_5_, (e) V_2_O_5_-ACNT1, (f) V_2_O_5_-ACNT3, and (g) V_2_O_5_-ACNT5, demonstrating the combined effects of lattice strain and crystal size. (h) Raman spectra of ACNT, V_2_O_5_, and V_2_O_5_-ACNT showing characteristic V–O stretching vibrations of V_2_O_5_ and D/G band features, (i) Enlarged Raman region highlighting variations in the D/G intensity ratio, confirming enhanced defect concentration and stronger interaction between V_2_O_5_ and ACNT with increasing ACNT content. (j) FTIR spectra of ACNT, V_2_O_5_, and V_2_O_5_-ACNT nanohybrids exhibiting typical bands assigned to V=O terminal stretching, V–O–V bridging modes, and oxygen-containing functionalities of ACNT.

Figure 4c shows the variation in crystallite size, lattice strain, and dislocation density of V_2_O_5_ and V_2_O_5_-ACNT nanohybrids. The crystallite size of V_2_O_5_ (∼23.6 nm) decreased for V_2_O_5_-ACNT3 (∼22 nm) as tabulated in Table 1, indicating interfacial strain and structural disorder induced by ACNT incorporation. This reduction was accompanied by higher lattice strain and dislocation density, further confirming the increased defect formation at the V_2_O_5_-carbon interface. This can be attributed to partial agglomeration of ACNTs and reduced effective interfacial contact, leading to localized relaxation of lattice strain. The controlled introduction of strain and defects is beneficial, as these defect-rich interfaces can act as active sites for hydroxyl radical generation, thereby enhancing pollutant removal efficiency. Figure 4d–g depict the Williamson-Hall (W–H) plots for V_2_O_5_ and V_2_O_5_-ACNT nanohybrids. Equation 1 is referred to as the W–H equation.

1 $$\beta \tau \;Cos\theta =\frac{0.9\lambda }{D}+4\varepsilon \;Sin\theta$$

**Table 1:** Structural Parameters (2θ, FWHM, Crystal Size, Lattice Strain, and Dislocation Density) of the Synthesized Samples (V_2_O_5_, V_2_O_5_-ACNT1, V_2_O_5_-ACNT3, and V_2_O_5_-ACNT5)

samples	2θ [°]	position [θ] [rad]	fwhm [rad]	crystal size [D] [nm]	Lattice strain [ε]	dislocation density [δ] [nm^–2^]
V_2_O_5_	20.3400	0.3550	0.36424	23.13396	0.00886	0.00187
V_2_O_5_-ACNT1	20.3496	0.3551	0.35405	23.80015	0.00861	0.00177
V_2_O_5_-ACNT3	20.3359	0.3549	0.38226	22.04327	0.00932	0.00206
V_2_O_5_-ACNT5	20.3428	0.3550	0.36949	22.80536	0.00899	0.00192

The positive slope of each linear fit indicates the presence of lattice strain, while the intercept corresponds to crystallite size.

Pristine V_2_O_5_ exhibits a moderate slope, reflecting limited lattice strain associated with well-ordered crystallites. In contrast, V_2_O_5_-ACNT3 exhibits a noticeably steeper slope, indicating greater lattice distortion and microstrain arising from strong interfacial interactions between ACNT and the V_2_O_5_ matrix. For V_2_O_5_-ACNT5, the slope decreases slightly, suggesting partial strain relaxation due to excessive ACNT loading, which may hinder uniform stress transfer across the interface and reduce the effective generation of defects.^38^ Raman spectra reveal characteristic V–O stretching modes of V_2_O_5_ alongside the D- and G-bands of ACNT, confirming chemical integration between the two components (Figure 4h,I). The variations in the D/G intensity ratio further confirmed the increased defect concentration and stronger interaction between V_2_O_5_ and ACNT with increasing ACNT content. Figure 4j further supports nanohybrid formation through FTIR analysis, displaying V=O terminal bonding, V–O–V bridging vibrations, and oxygen-containing functional groups from ACNT.

Field emission-scanning electron microscopy (FE-SEM) was employed to investigate the morphological evolution across the catalysts, ACNT, V_2_O_5_, and V_2_O_5_-ACNT nanohybrids. FE-SEM images of carbon nanotubes (CNTs) at different magnifications (Figure S6) reveal an entangled, interconnected CNT network, indicating good dispersion and high aspect ratios. The high-magnification image clearly shows a hollow, tubular structure with well-defined surfaces, confirming the characteristic morphology of CNTs and their suitability as a conductive and high-surface-area support material. FE-SEM images (Figure 5a–c and magnified views 5a_1_–c_1_) reveal distinct morphologies of the components and their hybrid structures. Pristine ACNT retains an interconnected, open-tubular network with uniformly distributed nanotubes (Figure 5a­(a_1_)). At higher magnification, the CNTs appear entangled within a porous scaffold that provides high-surface-area support and continuous electron pathways. Pristine V_2_O_5_ (Figure 5b­(b_1_)) exhibits the stacked platelet/flake morphology with well-defined edges and lateral dimensions with the order of tens to a hundred of nanometres, consistent with layered orthorhombic V_2_O_5_. In the V_2_O_5_-ACNT3 nanohybrids (Figure 5c­(c_1_)), V_2_O_5_ platelets are clearly anchored and wrapped around the ACNT network; the platelet fragments are smaller and more dispersed than in the pristine oxide, demonstrating effective nucleation of V_2_O_5_ on ACNT surfaces and suppression of large platelet aggregation.

**Figure 5:**
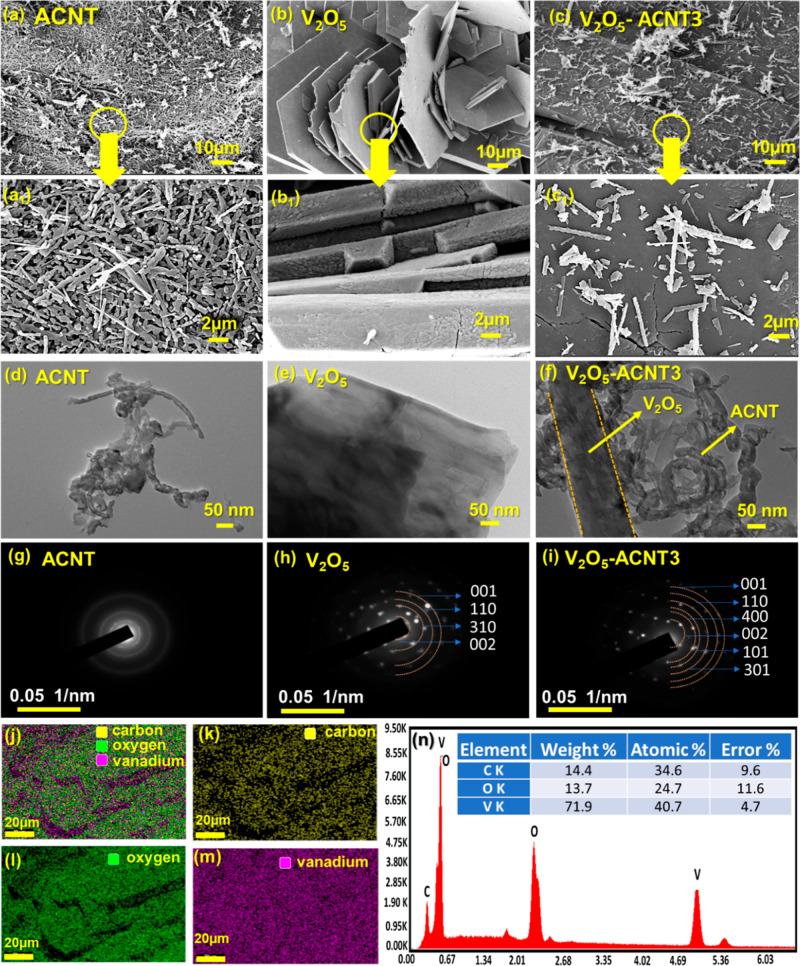
FE-SEM images of (a­(a1)) ACNT, (b­(b1)) V_2_O_5_, and (c­(c1)) V_2_O_5_-ACNT3, showing the uniform nanotubular network of ACNT, the stacked layered platelet morphology of V_2_O_5_, and the successful anchoring and dispersion of V_2_O_5_ particles onto ACNT surfaces in the hybrid system. (d) High-resolution TEM images of ACNT, (e) V_2_O_5_, and (f) V_2_O_5_-ACNT further confirm the interaction between CNTs and V_2_O_5_ nanosheets, revealing a well-integrated nanostructure. (g–i) SAED patterns showing a diffuse halo ring for ACNT, indicating its amorphous nature, sharp and well-defined diffraction spots corresponding to the crystalline planes of V_2_O_5_, and a hybrid ring-spot pattern for V_2_O_5_-ACNT confirming the coexistence of amorphous carbon nanotubes and crystalline V_2_O_5_, demonstrating enhanced crystallinity contributed by V_2_O_5_. (j–m) Elemental mapping of the V_2_O_5_-ACNT3, highlighting the homogeneous spatial distribution of carbon, oxygen, and vanadium throughout the hybrid. (n) EDX spectrum and quantitative elemental analysis confirming the presence and proportion of C, O, and V, verifying the successful formation of the V_2_O_5_-ACNT.

Additionally, the morphological features of V_2_O_5_-ACNT1 and V_2_O_5_-ACNT5 (Figures S7 and S8) show stacked, plate-like V_2_O_5_ structures with ACNTs sparsely yet uniformly decorating the platelet surfaces, indicating initial interfacial contact while largely preserving the pristine oxide morphology.

The V_2_O_5_-ACNT5 exhibits more pronounced surface roughening and fragmentation of V_2_O_5_ platelets, accompanied by dense anchoring and interwoven ACNT networks across the oxide surface. This evolution suggests that higher ACNT loading may increase nucleation sites and interfacial coupling, thereby improving V_2_O_5_ dispersion, increasing surface roughness, and promoting a more integrated hybrid architecture that facilitates efficient charge transport and reactive-site accessibility. HR-TEM images of ACNT, V_2_O_5_, and the V_2_O_5_-ACNT3 nanohybrid, respectively, further confirm this strong interfacial interaction (Figure 5d–f). ACNT exhibits a highly entangled tubular morphology with abundant structural defects, providing a large surface area and favorable anchoring sites. Pristine V_2_O_5_ exhibits well-defined layered nanosheets with smooth contrast, characteristic of its crystalline oxide nature. In the V_2_O_5_-ACNT3 composite, V_2_O_5_ nanosheets that are uniformly dispersed and associated with the ACNT network, indicating strong interfacial contact between the two components.

The corresponding SAED patterns (Figure 5g–i) further confirm these observations: ACNT displays a diffused halo ring, indicative of its largely amorphous carbon structure, while pristine V_2_O_5_ exhibits distinct diffraction rings indexed to the (001), (110), (310), and (002) planes, confirming its crystalline orthorhombic phase. Notably, the V_2_O_5_-ACNT3 composite retains the characteristic diffraction features of V_2_O_5_ with slight ring broadening, suggesting reduced crystallite size and increased lattice distortion induced by interfacial interaction with ACNT. This intimate interfacial contact and defect-rich structure are expected to facilitate charge transfer, enhance redox activity, and provide abundant active sites for H_2_O_2_ activation, thereby contributing to the superior photofenton catalytic performance of the V_2_O_5_-ACNT nanohybrids. Elemental mapping (Figure 5j–m) of V_2_O_5_-ACNT3 demonstrates a homogeneous spatial distribution of carbon, oxygen, and vanadium. The colocalization of V and O signals with the carbon framework confirms that V_2_O_5_ is uniformly dispersed over the ACNT surface rather than forming isolated agglomerated oxide clusters. The EDX spectrum and tabulated quantification (Figure 5n) corroborate these observations; the high vanadium content confirms the predominant incorporation of V_2_O_5_, whereas the noticeable carbon contribution arises from the ACNT matrix.

The optical and textural properties of ACNT, V_2_O_5_, and V_2_O_5_-ACNT nanohybrids were investigated using UV–visible spectroscopy to evaluate their light-harvesting capability and catalytic potential. Figure 6a shows the UV–vis absorption spectra of all samples recorded in the wavelength range of 200–1100 nm. Pristine V_2_O_5_ exhibits strong UV absorption, characteristic of charge-transfer transitions from O 2p to V 3d orbitals.^39^ In contrast, ACNT exhibits broad absorption extending into the near-visible region, originating from its conjugated carbon framework and defect-induced electronic states.^40^ Upon hybridization, V_2_O_5_-ACNT nanohybrids exhibited significantly enhanced and broadened absorption over the UV–visible region, with V_2_O_5_-ACNT3 and V_2_O_5_-ACNT5 showing the highest absorption intensities. This enhancement is attributed to synergistic interactions between V_2_O_5_ and ACNT, improved interfacial charge transfer, and defect-assisted light absorption.

**Figure 6:**
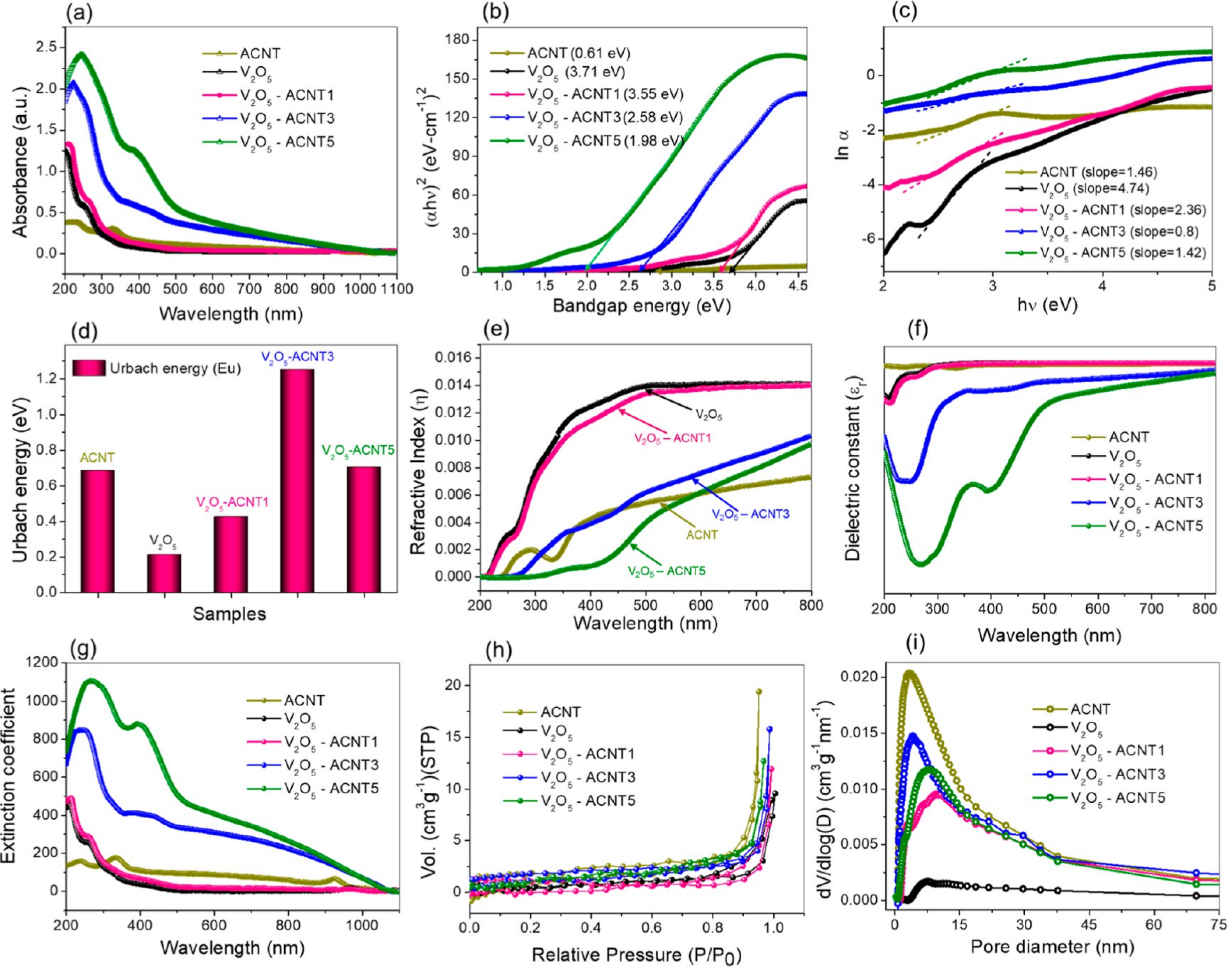
(a) UV–vis absorbance spectra of the catalyst, illustrating their optical absorption across the UV region. (b) Tauc plots (α*h*ν)^2^ versus *h*ν to estimate the optical bandgap, *E*_g_. (c) ln­(α) versus hν plots, (d) Urbach energy analysis showing increased structural disorder and tailing of localized states. (e) Refractive index, η, variation as a function of wavelength, (f) real part of the dielectric constant of the NCs demonstrating enhanced polarization response and stronger charge-carrier interaction within the hybrid structure, (g) extinction coefficient spectra for V_2_O_5_, V_2_O_5_-ACNT1, V_2_O_5_-ACNT3, and V_2_O_5_-ACNT5 catalyst, (h) N_2_ adsorption–desorption isotherm plot indicating mesoporous character of the catalyst, (i) pore size distribution plot for V_2_O_5_, V_2_O_5_-ACNT1, V_2_O_5_-ACNT3, and V_2_O_5_-ACNT5 catalyst.

The optical bandgap *E*_*g*_ of the samples (Figure 6b) was estimated from Tauc’s relation given by eq 2 by plotting (α*h*ν)^2^ versus photon energy (*h*ν)^41^

2 $${\left(\alpha hv\right)}^{i}=A\left(hv-E_{g}\right)$$

where α is the absorption coefficient, *A* is the band edge sharpness constant, and exponent *i* denotes the allowed electronic transitions (*i* = 2 for direct *E*_g_). The *E*_g_ was determined to be 0.61 eV for ACNT, 3.71 eV for V_2_O_5_, and 3.55, 2.58, and 1.98 eV for V_2_O_5_-ACNT1, V_2_O_5_-ACNT3, and V_2_O_5_-ACNT5, respectively. The progressive reduction in *E*_g_ upon ACNT incorporation indicates bandgap narrowing due to strong electronic coupling, defect-induced intermediate energy states, and enhanced orbital hybridization at the V_2_O_5_-carbon interface. To further examine structural disorder and defect states, the Urbach energy (*E*_u_) was also calculated (Figure 6c,d) from the exponential absorption edge using eq 3

3 $$\alpha =\alpha_{0}\exp\frac{\left(h\nu \right)}{\left(E_{u}\right)}orE_{u}=\frac{1}{slope}$$

V_2_O_5_-ACNT3 shows the highest *E*_u_ (Table S1), indicating the presence of controlled defect states and localized tail states near the band edges. These defect states play a crucial role in facilitating charge trapping, suppressing charge recombination, and enhancing catalytic efficiency. The wavelength-dependent refractive index (η) and dielectric constant (ε) were also evaluated using the absorption-derived optical constants. The refractive index was estimated using eqs 4 and 5, where, *n* is the refractive index, *A* and *T*_s_ are the absorbance and transmittance, respectively.

4 $$T_{s}=10^{\left(-A\right)}\times 100$$

5 $$n=\frac{1}{T_{s}}+\sqrt{\frac{1}{T_{s}-1}}$$

V_2_O_5_-ACNT nanohybrids exhibit higher refractive indices compared to pristine materials (Figure 6e), particularly in the visible region, consistent with enhanced light–matter interaction and reduced bandgap. The dielectric constant (Figure 6f) follows a similar trend, with V_2_O_5_-ACNT3 exhibiting a higher dielectric response, indicating improved polarization behavior and charge-storage capability. The extinction coefficient (*K*) spectra (Figure 6g) calculated using eq 6 further support the enhanced optical response of the V_2_O_5_-ACNT nanohybrids, particularly V_2_O_5_-ACNT3, which exhibit higher *K* values across the visible region, indicating increased photon absorption and stronger light–matter interaction. This enhanced optical behavior facilitates efficient photoexcitation and charge-carrier generation, thereby improving catalytic performance.

6 $$K=\frac{\alpha \lambda }{4\pi }$$

The N_2_ adsorption–desorption isotherms (Figure 6h) reveal that ACNT shows the highest adsorption capacity and a typical mesoporous behavior, whereas the addition of V_2_O_5_ reduces accessible pore volume (Table S2) due to partial filling of CNT pores by oxides. A pronounced rise in N_2_ uptake at high relative pressure (*P*/*P*_0_ > 0.8) suggests the presence of macropores and interparticle voids in the nanohybrids. The pore size distribution curves (Figure 6i) confirm mesoporous characteristics for all samples, with dominant pore diameters between ∼5 and 20 nm. Among the nanohybrids, V_2_O_5_-ACNT3 maintains a well-balanced pore structure, ensuring efficient diffusion pathways and abundant active sites.

Figure 7 collectively demonstrates the synergistic integration of V_2_O_5_ with ACNT, which noticeably enhances the photoinduced charge transport and reactive species (RS) generation. V_2_O_5_-ACNT3 nanohybrids exhibit a substantially higher and more stable photocurrent response than pristine V_2_O_5_ (Figure 7a), confirming efficient charge separation and rapid interfacial electron transport. This enhancement is further supported by the electron paramagnetic resonance (EPR) spectra (Figure 7b), where the V_2_O_5_-ACNT3 sample shows a more intense signal at *g* ≈2.00, indicating a higher concentration of surface defects, or oxygen vacancies, that serve as active catalytic sites. The radar plot (Figure 7c), compares the performance of V_2_O_5_-ACNT3 nanohybrids against various literature across five metrics, including efficiency, time, catalyst dosage, volume, and initial concentration. That confirms the superior efficiency even at higher pollutant concentrations compared to previously reported catalysts. To identify the primary drivers of this enhanced activity, electron spin resonance (ESR) measurements were conducted (Figure 7d–f). Under light irradiation, particularly in the presence of H_2_O_2_, characteristic signals for hydroxyl radicals (^•^OH), superoxide radicals ^•^O_2_^–^), and singlet oxygen (^1^O_2_) were observed. In addition to ^•^OH and ^•^O_2_^–^ radicals, ^1^O_2_ was also detected via ESR analysis, suggesting its auxiliary role in the oxidation process. ^1^O_2_ may participate in secondary oxidation pathways, further enhancing the overall catalytic performance. The marked increase in signal intensity in the “Light + H_2_O_2_” systems indicates a highly efficient radical-generation pathway, driven by a photofenton-like mechanism.

**Figure 7:**
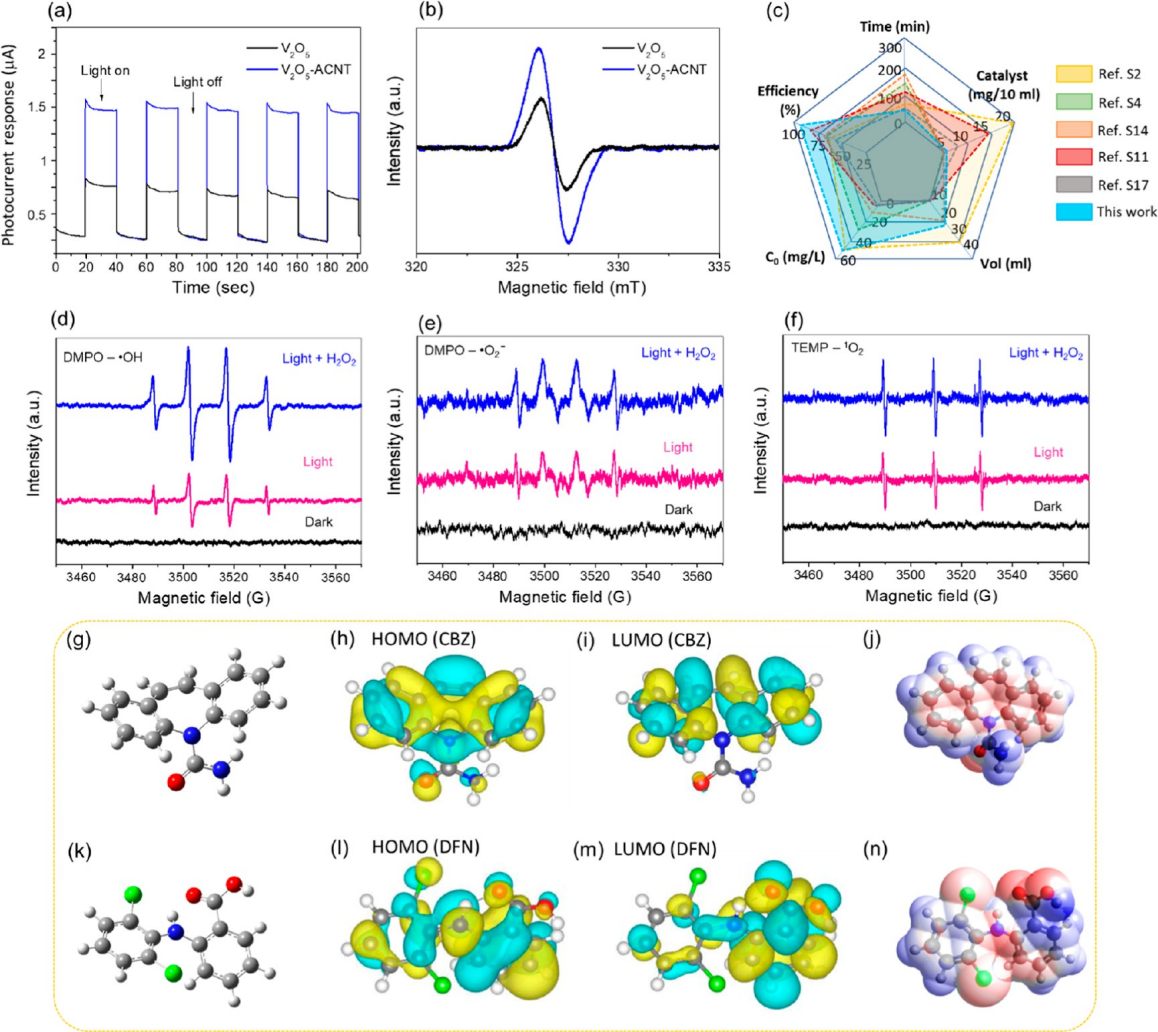
Photoelectrochemical and mechanistic analysis. (a) Transient photocurrent responses under periodic light on/off cycles, (b) EPR spectra highlighting oxygen vacancy concentrations, (c) Radar plot comparing catalytic performance metrics (efficiency, time, dosage, volume, and initial pollutant concentration) with reported literature, ESR spin-trapping spectra for (d) DMPO- ^•^OH, (e) DMPO- ^•^O_2_^–^, and (f) TEMP-^1^O_2_ under dark, light, and light + H_2_O_2_ conditions, (g–j) DFT optimized CBZ structure with corresponding HOMO, LUMO and ESP mapping respectively, (k–n) DFN: DFT-optimized structure, HOMO, LUMO, and ESP distribution, respectively.

Theoretical investigation further elucidates the molecular-level interaction and removal susceptibility of target pollutants. The DFT-optimized geometries of CBZ and DFN (Figure 7g,k) reveal stable conformations with exposed heteroatoms and aromatic domains that act as potential adsorption and reaction sites on the catalyst surface. The HOMO distributions (Figure 7h,l) are primarily localized over the conjugated aromatic rings and amine/heteroatom regions, indicating electron-rich centers that are highly prone to oxidative attack by photogenerated ^•^OH and ^1^O_2_ species.^42^ In contrast, the LUMO maps (Figure 7i,m) show electron-deficient regions concentrated near carbonyl and halogen-substituted groups, facilitating electrophilic interactions and promoting electron acceptance during redox processes. The electrostatic potential (ESP) surfaces (Figure 7j, n) further distinguish nucleophilic (negative, red) and electrophilic (positive, blue) domains, highlighting preferential adsorption sites for reactive oxygen species and H_2_O_2_ activation. Notably, DFN exhibits stronger charge polarization due to electron-withdrawing substituents, suggesting higher susceptibility toward radical-induced fragmentation, while CBZ shows delocalized electron density that favors initial hydroxylation followed by ring opening.

X-ray photoelectron spectroscopy (XPS) was employed to elucidate the surface chemical composition and electronic states of ACNT, V_2_O_5_, and V_2_O_5_-ACNT nanohybrids (Figure 8a–i). Binding energies were calibrated with respect to the C 1s reference peak. The full survey spectra (Figure 8a) confirm the coexistence of C, O, and V in the samples, thereby verifying the successful integration of V_2_O_5_ into ACNT. Also, the absence of impurity-related peaks indicates high chemical purity of the synthesized materials. The C 1s spectrum of ACNT (Figure 8b) exhibits the dominant C 1s feature at ∼282.0 eV, attributed to C–C bonding related to the carbon network of ACNT,^43^ along with additional components associated with C–O–C and O–C=O functionalities, confirming surface activation and defect formation. These oxygenated carbon species serve as electron-rich sites, facilitating interfacial coupling with V_2_O_5_. Upon hybridization, the C 1s spectra of V_2_O_5_-ACNT nanohybrids (Figure 8e,g,i) show increased intensity of carbon species, indicating enhanced interfacial bonding and electronic interaction between the carbon framework and V_2_O_5_. The O 1s core-level spectra of V_2_O_5_ (Figure 8c) can be deconvoluted into lattice oxygen (V–O–V) at ∼530.0 eV and surface hydroxyl groups at higher binding energies, reflecting the intrinsic oxide framework. In V_2_O_5_-ACNT nanohybrids, the relative contribution of surface –OH species increases noticeably, suggesting the formation of oxygen vacancies and defect-rich environments induced by ACNT incorporation, which are known to facilitate redox-active behavior and reactive oxygen species (ROS) generation. Crucially, the V 2p spectra provide insight into the oxidation state of vanadium. For V_2_O_5_ (Figure 8c), the V 2p_3/2_ and V 2p_1/2_ peaks centered at ∼517.0 and ∼524.5 eV correspond predominantly to V^5+^ species. In the V_2_O_5_-ACNT nanohybrids (Figure 8d,f,h), these peak positions remain largely unchanged. However, the increased contribution of surface –OH species in the O 1s spectra, together with subtle peak broadening and asymmetry in the V 2p envelope, suggests a modified local electronic environment of vanadium arising from strong interfacial interactions and charge redistribution between V_2_O_5_ and the conductive ACNT network. Such electronic perturbations can facilitate reversible V^5+^/V^4+^ redox transitions under operational photofenton conditions. This interpretation is consistent with DFT-predicted interfacial charge transfer and correlates well with the enhanced catalytic performance observed for the V_2_O_5_-ACNT nanohybrids. Overall, the XPS results indicate that ACNT effectively modulates the surface electronic structure of V_2_O_5_ and promotes the formation of redox-active vanadium centers, thereby supporting efficient H_2_O_2_ activation during photofenton reactions.

**Figure 8:**
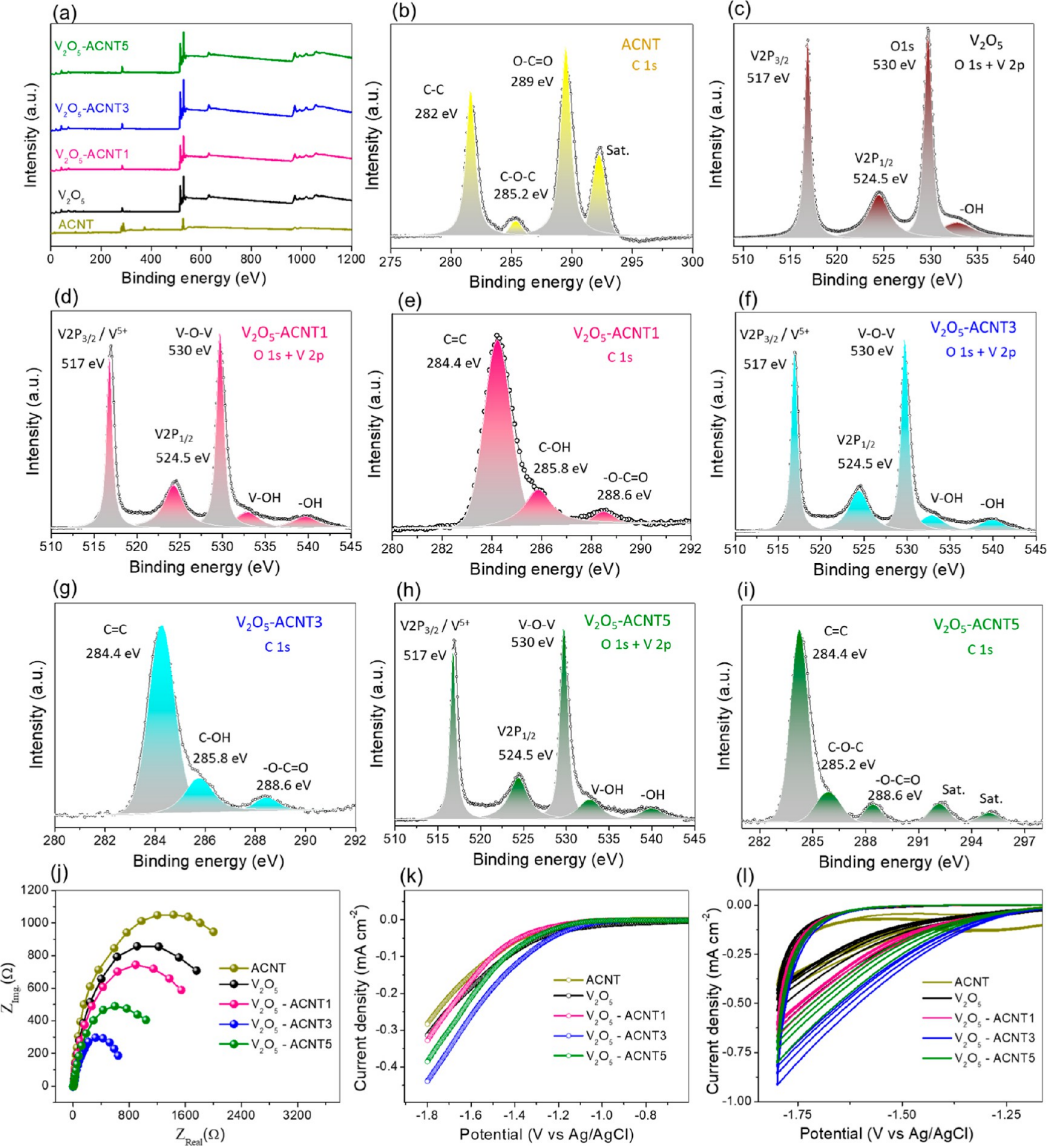
(a) The XPS-full survey spectra of catalyst ACNT, V_2_O_5_, V_2_O_5_-ACNT1, V_2_O_5_-ACNT3, and V_2_O_5_-ACNT5, (b) C 1s spectrum for ACNT, (c,d) O 1s and V 2p spectrum of V_2_O_5_ and V_2_O_5_-ACNT1 respectively, (e) C 1s spectrum for V_2_O_5_-ACNT1, (f) O 1s and V 2p spectrum of V_2_O_5_-ACNT3, (g) C 1s spectrum for V_2_O_5_-ACNT3, (h) O 1s and V 2p spectrum of V_2_O_5_-ACNT5, (i) C 1s spectrum for V_2_O_5_-ACNT5, (j–l) EIS plot, CV curve and LSV plot for ACNT, V_2_O_5_, V_2_O_5_-ACNT1, V_2_O_5_-ACNT3, and V_2_O_5_-ACNT5, respectively.

Complementary electrochemical measurements further corroborate ACNT’s role in modulating interfacial charge-transfer behavior. The electrochemical impedance spectra (Figure 8j) reveal a pronounced reduction in the semicircle diameter for the V_2_O_5_-ACNT nanohybrids compared to pristine V_2_O_5_, indicating a significantly lower charge-transfer resistance at the electrode–electrolyte interface. Among all samples, V_2_O_5_-ACNT3 exhibits the smallest impedance arc, demonstrating the most efficient interfacial electron transport facilitated by the conductive ACNT network. The transient photocurrent responses (Figure 8k) show markedly enhanced, stable photocurrent densities for the V_2_O_5_-ACNT composites under illumination, with V_2_O_5_-ACNT3 exhibiting the highest photocurrent intensity, reflecting rapid photoinduced charge separation and suppressed electron–hole recombination. Furthermore, the cyclic voltammetry (CV) curves (Figure 8l) show higher current responses and larger enclosed areas for the V_2_O_5_-ACNT nanohybrids than for pristine V_2_O_5_, indicating enhanced electrochemical activity and faster interfacial electron-transfer processes. These electrochemical features are fully consistent with the XPS-derived surface electronic modulation and DFT-predicted charge redistribution, collectively confirming that ACNT incorporation establishes an efficient electron-transport framework that supports rapid vanadium redox turnover and sustained H_2_O_2_ activation during photofenton reactions.

### Performance Evaluation in Mixed Pollutant Systems

The defect chemistry at the V_2_O_5_-ACNT interface plays a crucial role in its catalytic performance, where oxygen vacancies induce localized V^4+^ sites that efficiently anchor H_2_O_2_. This interaction facilitates the rapid breakdown of H_2_O_2_ into highly reactive hydroxyl radicals (^•^OH), driving the surface reaction (Figure S9). The concentrations of CBZ and DFN were quantified using UV–vis spectroscopy at their respective maximum absorption wavelengths (λ_max_), 285 nm for CBZ and 276 nm for DFN. Calibration curves were constructed using a series of standard solutions of known concentrations, exhibiting good linearity (Figure S10). The high linear correlation between absorbance and concentration ensures reliable quantification of pollutant concentrations during the removal process. Accordingly, the removal efficiency was calculated based on the relative concentration changes derived from the corresponding absorbance values. The individual removal behavior of CBZ and DFN, along with their corresponding kinetic parameters under identical reaction conditions, was confirmed (Figures S11–S14), with the superior activity and fastest removal kinetics of the V_2_O_5_-ACNT3 catalyst compared to pristine V_2_O_5_ and other ACNT loadings. Figure 9a,d present the time-dependent catalytic absorbance profiles of CBZ and DFN mixed pollutants over pristine V_2_O_5_ and V_2_O_5_-ACNT nanohybrids under direct solar irradiation. Compared to pristine V_2_O_5_, all nanohybrids exhibit higher removal kinetics, with V_2_O_5_-ACNT3 showing the highest removal efficiency, indicating superior catalytic performance. Figure 9e depicts the relative concentration (*C*_t_/*C*_0_) of mixed pollutants versus time (min) concerning different catalysts, indicating the maximum removal in 60 min for V_2_O_5_-ACNT3. During the light-off state, no significant pollutant removal was observed due to the absence of photon energy, whereas the catalytic process was initiated under the light-on state. The pseudo-first-order kinetic plots (Figure 9f,g), estimated using eq 7,^44^ were employed to evaluate the pollutant removal rate (k) for each sample.

7 $$\ln\frac{C_{o}}{C_{t}}=k\cdot t\;\text{or}\;C_{o}=C_{t}\left[\exp\left(k\cdot t\right)\right]$$

**Figure 9:**
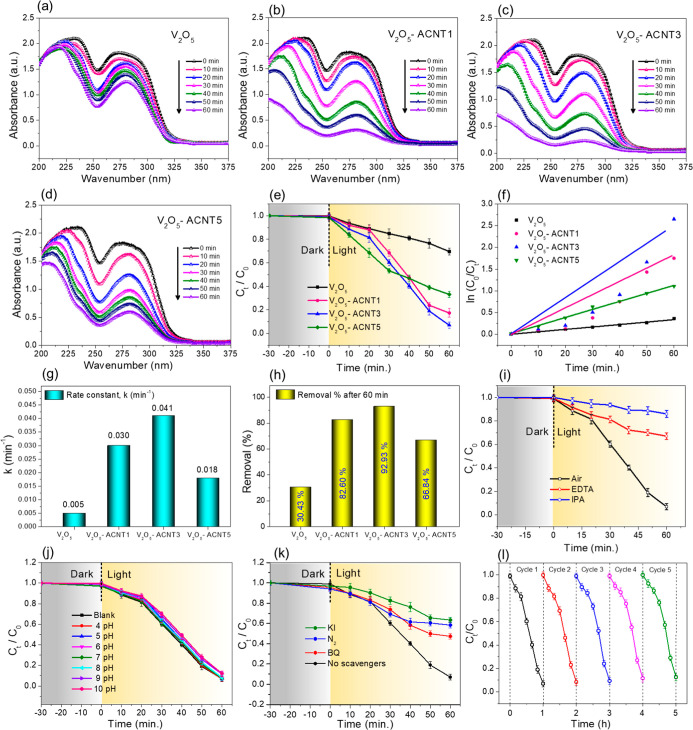
(a–d) Time-dependent catalytic removal profiles of mixed pollutants (CBZ and DFN) under the solar light illumination using V_2_O_5_, V_2_O_5_-ACNT1, V_2_O_5_-ACNT3, and V_2_O_5_-ACNT5 catalysts, respectively. (e) Relative concentration (*C*_t_/*C*_0_) of mixed pollutant as a function of time (min) with different catalysts. (f) Pseudo-first-order kinetic plots: ln­(*C*_0_/*C*_t_) versus time, (g) removal rate constant demonstrating the highest activity for V_2_O_5_-ACNT3 (h) Catalytic removal efficiency (%) of mixed pollutant within 60 min of solar irradiation, (i) effect of atmospheric conditions (Air, EDTA, and IPA) on removal efficiency from the mixed pollutant system using V_2_O_5_-ACNT3 catalyst, (j) Influence of initial solution pH on the catalytic removal efficiency of V_2_O_5_-ACNT3 catalyst. (k) Reactive oxygen species (ROS) trapping experiments using KI, N_2_, and BQ to identify dominant oxidative pathways. (l) Reusability study of the V_2_O_5_-ACNT3 catalyst over consecutive catalytic cycles, confirming structural stability and sustained performance.

Where *C*_0_ is the starting concentration, *C*_t_ is the concentration at a time “*t*”, and *k* is the rate constant. The computed rate constants values (Table 2) showed the maximum removal rate for the V_2_O_5_-ACNT3 (0.041 min^–1^) and detected the highest removal efficiency (92.93%) against the mixed pollutant system (Figure 9h), underscoring the optimized ACNT loading for enhanced catalytic activity, due to improved light absorption, efficient charge separation, and rapid interfacial electron transport enabled by the conductive ACNT network. To assess the influence of RS on the catalytic activity, experiments were conducted under different atmospheric and scavenging conditions (Figure 9i). The removal efficiency decreases markedly in the presence of EDTA, indicating that photogenerated holes (h^+^) contribute to the removal process by facilitating the formation of RS. A sharp decrease in catalytic activity was observed upon the addition of IPA, confirming the role of ^•^OH radicals in sustaining efficient catalytic activity.

**Table 2:** Rate Constant and the Removal Rate for the Samples

samples	rate (min^–1^)			removal rate (%)		
	CBZ	DFN	Mix	CBZ	DFN	Mix
V_2_O_5_	0.011	0.006	0.005	47.28	33	30.43
V_2_O_5_-ACNT1	0.026	0.021	0.030	76.08	73.5	82.60
V_2_O_5_-ACNT3	0.053	0.055	0.041	95.10	96	92.93
V_2_O_5_-ACNT5	0.014	0.022	0.018	60.32	71	66.84

The effect of solution pH on catalytic removal is shown in Figure 9j. The removal efficiency increases from acidic to near-neutral conditions and reaches a maximum around pH 7.0–8.0, reflecting favorable surface charge interactions and enhanced ROS generation. However, further increasing the pH to alkaline conditions (pH 9–10) results in a gradual decline in removal efficiency, likely due to electrostatic repulsion between negatively charged catalyst surfaces and pollutant molecules, as well as scavenging of ^•^OH radicals by excess OH^–^ ions. RS trapping experiments (Figure 9k) further reveal that the removal efficiency decreases in the presence of Benzoquinone (BQ) and N_2_, confirming that ^•^O_2_^–^ radicals are also crucial RS responsible for pollutant removal. The moderate suppression observed with KI indicates that photogenerated h^+^ also indirectly facilitates the RS formation. To further quantify the relative contribution of these RS in the removal process, the contribution percentage of each radical was estimated using eq 8.

8 $$contribution\;\left(\%\right)=\frac{{removal}_{\left(control\right)}-{removal}_{\left(scavenger\right)}}{{removal}_{\left(control\right)}}\times 100$$

where removal _(control)_ represents the removal efficiency in the absence of scavengers (air condition), and removal _(scavenger)_ corresponds to the removal efficiency in the presence of specific radical scavengers. For CBZ, the removal efficiency decreased from 95.10% (control) to 25.93% and 58.47% in the presence of IPA and BQ, respectively. This corresponds to ^•^OH and •O_2_^–^ contributions of 72.73% and 38.51%, indicating that •OH radicals are the dominant oxidative species. Similarly, for DFN, the removal efficiency decreased from 96% (control) to 21.47% and 51.28% in the presence of IPA and BQ, respectively. The calculated contributions of ^•^OH and ^•^O_2_^–^ are 77.61% and 46.63%, respectively, further confirming the predominant involvement of ^•^OH radicals, with a secondary contribution from ^•^O_2_^–^ in the removal process. This quantitative analysis further corroborates the ESR results and confirms the dominance of the photo-Fenton-driven ^•^OH pathway in the V_2_O_5_-ACNT system. The recyclability and durability of V_2_O_5_-ACNT3 were examined over five successive catalytic cycles (Figure 9l). The catalyst retains removal efficiencies above 90% throughout the cycles, indicating high structural integrity and strong resistance to photocorrosion.

To further validate the optimized ACNT loading, an intermediate composition (V_2_O_5_-ACNT4) was additionally evaluated (Figure S15). Although V_2_O_5_-ACNT4 exhibits appreciable mixed-pollutant removal (∼82–85% within 60 min) and follows pseudo-first-order kinetics (*k* ≈0.017 mim^–1^), its activity remains inferior to that of V_2_O_5_-ACNT3. The comparatively lower performance of V_2_O_5_-ACNT4 suggests that excessive ACNT content partially shields the active V_2_O_5_ sites, and thereby weakens effective light utilization and interfacial charge transfer. This sustained performance highlights the robustness and reusability of the optimized V_2_O_5_-ACNT3 nanohybrid under solar irradiation. Moreover, its consistent efficiency in simultaneously removing CBZ and DFN underscores its practical potential for treating mixed pharmaceutical contaminants in real wastewater systems.

### Photofenton Removal Mechanism

The enhanced photofenton removal activity of the V_2_O_5_-ACNT nanohybrids originates from the synergistic integration of V_2_O_5_ with the conductive, defect-rich ACNT network, which together promotes efficient charge separation, interfacial electron transfer, and sustained V^5+^/V^4+^ redox cycling. A schematic representation of the proposed mechanism is illustrated in Figure 10a. Upon solar irradiation, V_2_O_5_ absorbs photons and generates electron–hole pairs due to its semiconductor nature (eq 9).^45^

9 $$V_{2}O_{5}+hv\to e_{CB}^{-}+h_{VB}^{+}$$

**Figure 10:**
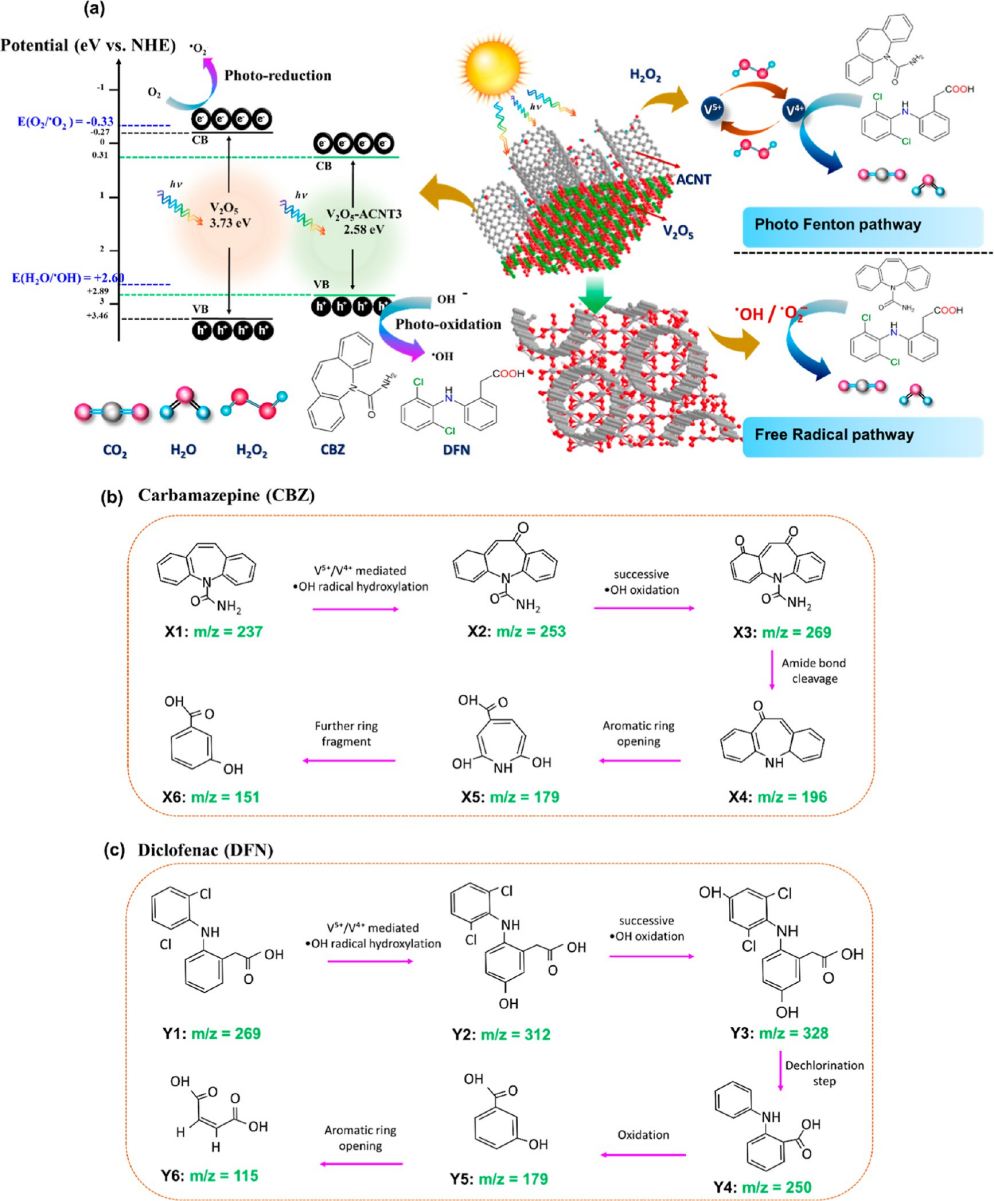
(a) Schematic illustration of the proposed catalytic photofenton removal mechanism over V_2_O_5_-ACNT nanohybrids, (b,c) Catalytic removal pathway of CBZ and DFN by V_2_O_5_-ACNT Nanohybrids as catalyst.

The incorporation of ACNT significantly narrows the effective bandgap and introduces conductive pathways, as confirmed by UV–vis and DFT analyses, enabling rapid migration of photogenerated electrons from the V_2_O_5_ conduction band to the ACNT network (eq 10). This electron extraction suppresses bulk recombination, which is consistent with the enhanced photocurrent response and reduced charge-transfer resistance observed in electrochemical measurements.

10 $$e_{CB}^{-}\left(V_{2}O_{5}\right)\to e^{-}\left(ACNT\right)$$

The transferred electrons reduce surface-adsorbed molecular oxygen to generate superoxide radicals, eq 11.

11 $$O_{2}+e^{-}\to \cdot O_{2}^{-}$$

Simultaneously, photogenerated holes participate in surface oxidation reactions, eqs 12 and 13, either directly contributing to pollutant removal or reacting with surface hydroxyl groups and water molecules to form hydroxyl radicals

12 $$h_{VB}^{+}+H_{2}O\to^{\cdot }OH+H^{+}$$

13 $$h_{VB}^{+}+{OH}^{-}\to^{\cdot }OH$$

A key feature of the V_2_O_5_-ACNT system is the vanadium photo-Fenton redox cycle, which is facilitated by interfacial charge redistribution, as evidenced by XPS-derived electronic perturbations and DFT-predicted charge transfer. In the presence of H_2_O_2_, surface V^5+^ species are reduced to V^4+^, accompanied by the generation of hydroxyl radicals, eq 14.

14 $$V^{5+}+H_{2}O_{2}\to V^{4+}{+}^{\cdot }OH+{OH}^{-}$$

The reduced V^4+^ species are rapidly reoxidized back to V^5+^ via reactions with H_2_O_2_ or dissolved oxygen, eqs 15 and 16, thereby completing the redox cycle.

15 $$V^{4+}+H_{2}O_{2}\to V^{5+}{+}^{\cdot }OH+{OH}^{-}$$

16 $$V^{4+}+O_{2}\to V^{5+}{+}^{\cdot }O_{2}^{-}$$

This continuous V^5+^/V^4+^ cycling acts as an efficient electron-shuttling system, sustaining the production of highly reactive oxygen species (^•^OH and ^•^O_2_^–^) under solar irradiation. The presence of surface –OH groups and oxygen-rich functional sites, identified from O 1s XPS spectra, further enhances H_2_O_2_ activation and stabilizes the redox-active vanadium centers. The generated reactive species attack CBZ and DFN molecules adsorbed on the catalyst surface via photofenton pathways, eq 17, leading to progressive bond cleavage and mineralization into benign products such as CO_2_ and H_2_O.

17 $$\cdot OH/\cdot O_{2}^{-}+CBZ/DFN\to intermediates+{CO}_{2+}+H_{2}O$$

Scavenger experiments corroborate this mechanism by demonstrating the contribution of RS species.

The superior activity of V_2_O_5_-ACNT3 is attributed to its optimized interfacial contact, balanced defect density, and maximized redox accessibility, which enables rapid and sustained photofenton removal under solar irradiation. The transformation pathways of CBZ and DFN during photofenton removal over V_2_O_5_-ACNT nanohybrids were further elucidated by LC–MS analysis (Figure 10b,c). The LC–MS analysis was performed using electrospray ionization in positive mode (ESI^+^) for CBZ and negative mode (ESI^–^) for DFN, selected based on their molecular characteristics and ionization behavior. CBZ undergoes initial ^•^OH-mediated hydroxylation to form Oxcarbazepine (*m*/*z* ≈253) (Figure S16 and Table S3), followed by successive oxidative steps yielding a quinone derivative of oxcarbazepine intermediates (*m*/*z* ≈266). Subsequent amide bond cleavage and aromatic ring–opening reactions generate lower-molecular-weight products, including anthranilic acid and hydroxy-benzoic acid derivatives, indicating progressive molecular fragmentation. Similarly, DFN transformation pathways identified from MS spectra (Figure S17 and Table S4) reveal stepwise hydroxylation to mono- and dihydroxy-diclofenac intermediates (*m*/*z* ≈312 and 328), followed by C–N bond cleavage and aromatic ring scission, ultimately producing low-molecular-weight carboxylic acids, such as maleic acid (*m*/*z* ≈115). The progressive breakdown of aromatic structures into smaller, low-molecular-weight intermediates indicates a systematic reduction in molecular complexity and a clear shift toward advanced oxidation. The formation of oxygenated fragments, such as hydroxy-substituted and carboxylic acid derivatives, suggests continuous oxidative cleavage of stable aromatic rings, ultimately reflecting a strong tendency toward effective mineralization of the parent pollutants.

The catalytic removal performance of V_2_O_5_-ACNT nanohybrids was also compared with reported catalysts in the literature (Table S5). Overall, this study demonstrates that V_2_O_5_-ACNT nanohybrids effectively regulate charge transfer and V^5+^/V^4+^ redox cycling, enabling efficient solar-driven photofenton removal of mixed pharmaceutical contaminants. The mechanistic insights and performance benchmarks presented herein provide a rational framework for designing next-generation redox-active catalysts for sustainable wastewater remediation. It is important to note that this study was conducted under well-stirred conditions to minimize mass transfer limitations and evaluate intrinsic catalytic activity. In practical applications, reduced mixing may introduce diffusion constraints for H_2_O_2_ and pollutants. However, the high surface area and strong adsorption affinity toward pollutants and H_2_O_2_ can facilitate localized concentration and enhance interfacial reactions. Additionally, the continuous generation of reactive species via V^5+^/V^4+^ redox cycling is expected to sustain catalytic activity even under less ideal mixing conditions.

## Conclusions

V_2_O_5_-ACNT nanohybrids were successfully prepared to achieve efficient solar-driven photofenton removal of mixed pharmaceutical pollutants (CBZ and DFN), with the optimized V_2_O_5_-ACNT3 catalyst delivering the highest performance. Under solar irradiation, V_2_O_5_-ACNT3 attained a removal efficiency of ∼92% within 60 min for the mixed-pollutant system. The apparent pseudo-first-order rate constant reached ∼0.041 min^–1^, representing an ∼8-fold enhancement compared to bare V_2_O_5_ (∼0.005 min^–1^), thereby confirming substantially accelerated removal kinetics. DFT simulations identified interfacial H_2_O_2_ adsorption as the key initiating step of the photofenton process on the V_2_O_5_-ACNT surface. In particular, dissociative adsorption of H_2_O_2_ at V–O interfacial sites was found to be energetically most favorable, accompanied by pronounced charge redistribution from the conductive ACNT framework to surface vanadium centers. This interfacial charge transfer, supported by pronounced charge redistribution and the thermodynamically favorable Gibbs free-energy landscape for H_2_O_2_ activation, stabilizes redox-active vanadium sites and thereby facilitates dynamic V^5+^/V^4+^ cycling under operational conditions. These theoretical insights are fully consistent with experimental XPS and electrochemical analyses, which revealed a modified vanadium electronic environment and enhanced interfacial charge transport in the nanohybrids. The synergistic coupling between V_2_O_5_ and ACNT promotes efficient charge separation, sustained V^5+^/V^4+^ redox cycling, and continuous activation of reactive species, collectively accounting for the superior photofenton removal performance. Overall, this study establishes a clear structure-property-activity relationship, highlighting interfacial redox chemistry in V_2_O_5_-ACNT nanohybrids as a robust and practical strategy for solar-driven removal of mixed pharmaceutical contaminants from wastewater systems.

## Supplementary Material



## Notes

Effect of catalyst dosage and H_2_O_2_ concentration on mixed pollutant removal; DFT structures and potential distributions of CNT, ACNT and V_2_O_5_-ACNT; DFT-optimized CBZ intermediates and ESP maps; DFT-optimized DFN intermediates and ESP maps; FESEM images of CNTs, V_2_O_5_-ACNT1 and V_2_O_5_-ACNT5; DFT mechanism of H_2_O_2_ activation and ^•^OH generation; CBZ removal performance; CBZ kinetics and rate analysis; DFN removal performance; DFN kinetics and rate analysis; performance of V_2_O_5_-ACNT4 catalyst; MS analysis of CBZ intermediates; MS analysis of DFN intermediates (PDF)
