# Supplementary material for: Interfacial V5+/V4+ Redox Kinetics Enabling Fe-free Photo-Fenton Catalysis in α‑V2O5‑ACNT Nanohybrids for Efficient Removal of Mixed CBZ and DFN
Source: ACS Appl Mater Interfaces. 2026 Apr 15;18(16):23345–63. doi: 10.1021/acsami.6c04932 (PMC13133796; doi:10.1021/acsami.6c04932)
Supplement: Supplementary file 1 [file am6c04932_si_001.pdf]

Supporting Information

Interfacial V^{5+}/V^{4+} Redox Kinetics Enabling Fe-free Photo-Fenton Catalysis in α-V_2O_5-ACNT Nanohybrids for Efficient Removal of Mixed CBZ and DFN

Deepak Kumar[†], Manisha Sharma[†], Sangeeta Shukla[†], Marshal Dhayal[‡], Ajeet Kumar Kaushik[§]*, Sanjeev Kumar Sharma[†]*

[†]Biomaterials and Sensor Laboratory, Department of Physics, Chaudhary Charan Singh University, Meerut, Uttar Pradesh, 250004, **India**

[‡]Laboratory of Plasma Processing and Biophysics, Department of Physics, Indian Institute of Technology (IIT) Delhi, New Delhi 110016, **India**

[§]NanoBioTech Laboratory, Department of Chemistry, Florida Polytechnic University, Lakeland, FL 33805, **USA**

*Corresponding authors: akaushik@floridapoly.edu (AK), sksharma18@ccsuniversity.ac.in (SKS)

Supporting contents:

Figure S1. (a) Relative concentration (C_t/C_0) of mixed pollutant (CBZ and DFN) as a function of irradiation time (min) at different dosages of the V_2O_5-ACNT3 catalyst, **(b)** Relative concentration (C_t/C_0) of mixed pollutant as a function of irradiation time (min) at varying H_2O_2 concentration using 5 mg of V_2O_5-ACNT3 catalyst.

Figure S2. DFT-simulated atomic structure models of **(a)** pristine carbon nanotube (CNT), **(b)** effective potential distribution of CNT, **(c)** activated carbon nanotube (ACNT) with surface oxygen-containing functional groups, and **(d)** corresponding effective potential distribution of ACNT.

Figure S3. DFT-simulated atomic structure model of **(a)** V_2O_5-ACNT showing octahedral coordination (green polyhedra) representing active catalytic sites, **(b)** effective potential distribution of V_2O_5-ACNT highlighting inner low-potential regions responsible for charge localization and preferential reactant adsorption.

Figure S4. (a-e) DFT-optimized successive removal intermediates of CBZ, **(a1-e1)** Corresponding electrostatic surface potential (ESP) maps highlighting reactive sites for radical attack.

Figure S5. (a) DFT-optimized successive removal intermediates of DFN, **(a1-e1)** Corresponding ESP distributions illustrating charge redistribution and preferential bond-cleavage regions.

Figure S6. FESEM images of CNTs: **(a)** Entangled CNT network at a magnification of 2 µm, **(b)** High-magnification image at 200 nm, highlighting the tubular morphology and surface texture of individual CNTs.

Figure S7. FESEM images of V_2O_5-ACNT1: **(a)** Stacked, plate-like V_2O_5 structures decorated with CNTs, **(b)** High-magnification image revealing the surface morphology and uniform distribution of CNTs on the V_2O_5 plates.

Figure S8. FESEM images of V_2O_5-ACNT5: **(a)** Layered V_2O_5 plate morphology with surface roughening, **(b)** High-magnification image highlighting the dense anchoring and interwoven distribution of CNTs on the V_2O_5 surface.

Figure S9. (a) Initial V_2O_5-ACNT interface showing V^{5+} states, **(b)** Creation of an oxygen vacancy resulting in localized V^{4+} sites, **(c)** Interaction of H_2O_2 molecule at the V_2O_5-ACNT interface, **(d)** Generation of a hydroxyl radical (•OH) at the surface.

Figure S10. Calibration curves of **(a)** CBZ at 285 nm and **(b)** DFN at 276 nm, showing linear correlation between absorbance and concentration for quantitative analysis.

Figure S11. Photocatalytic removal of CBZ under solar light irradiation within 60 min by catalysts, **(a)** V_2O_5, **(b)** V_2O_5-ACNT1, **(c)** V_2O_5-ACNT3, **(d)** V_2O_5-ACNT5.

Figure S12. (a) Relative concentration (C_t/C_0) of CBZ with respect to time (min) in light-off/-on states, **(b)** first-order kinetic graphs, $ln(C_0/C_t)$ versus time (min), **(c)** removal rate constant demonstrating the highest activity for V_2O_5-ACNT3 against CBZ, **(d)** CBZ removal % by catalysts, **(e-f)** Effect of radical scavengers (BQ and IPA) on CBZ removal efficiency after 60 min, indicating the role of $•O_2^-$ and •OH species.

Figure S13. Photocatalytic removal of DFN under solar light irradiation within 60 min by catalysts, **(a)** V_2O_5, **(b)** V_2O_5-ACNT1, **(c)** V_2O_5-ACNT3, **(d)** V_2O_5-ACNT5.

Figure S14. (a) Relative concentration (C_t/C_0) of DFN with respect to time (min) in light-off/-on states, **(b)** first-order kinetic graphs, *ln*(C_0/C_t) versus time (min), **(c)** removal rate constant demonstrating the highest activity for V_2O_5-ACNT3 against DFN, **(d)** DFN removal % by catalysts, **(e-f)** Effect of radical scavengers (BQ and IPA) on DFN removal efficiency after 60 min, indicating the role of $\bullet O_2^-$ and $\bullet OH$ species.

Figure S15. (a) Photocatalytic removal of mixed pollutants (CBZ and DFN) under solar light irradiation within 60 min using V_2O_5-ACNT4 catalyst, **(b)** relative concentration (C_t/C_0) of mixed pollutant as a function of time (min) with catalyst V_2O_5-ACNT4, **(c)** Pseudo-first-order kinetic plots: ln(C_0/C_t) versus time, **(d)** Photocatalytic removal efficiency (%) of mixed pollutant within 60 min of solar irradiation by V_2O_5-ACNT4.

Figure S16. MS results of CBZ intermediate products.

Figure S17. MS results of DFN intermediate products.

Table S1. The values of bandgap (E_g) and Urbach energy (E_u) for prepared samples (ACNT, V_2O_5, V_2O_5-ACNT1, V_2O_5-ACNT3, and V_2O_5-ACNT5).

Table S2. Average pore size, surface area, and pore volume of (ACNT, V_2O_5, V_2O_5-ACNT1, V_2O_5-ACNT3, and V_2O_5-ACNT5) catalysts.

Table S3. CBZ removal products by mass spectra results.

Table S4. DFN removal products by mass spectra results.

Table S5. Comparison of the catalytic performance of V_2O_5-ACNT Nanohybrids with others catalysts.

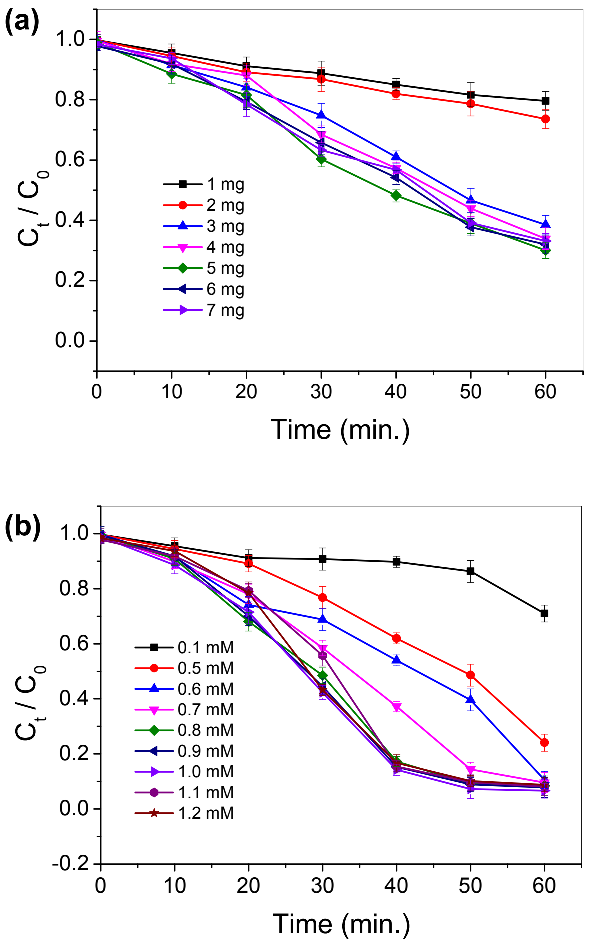

Figure S1. (a) Relative concentration (C_t/C_0) of mixed pollutant (CBZ and DFN) as a function of irradiation time (min) at different dosages of the V_2O_5-ACNT3 catalyst, **(b)** Relative concentration (C_t/C_0) of mixed pollutant as a function of irradiation time (min) at varying H_2O_2 concentration using 5 mg of V_2O_5-ACNT3 catalyst.

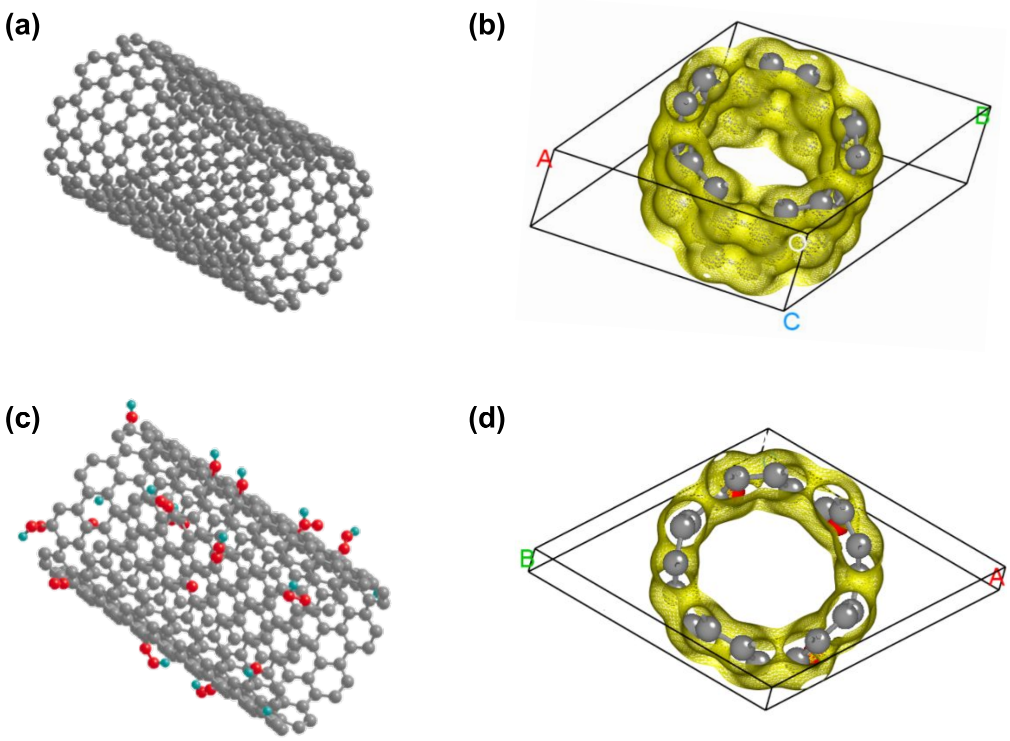

Figure S2. DFT-simulated atomic structure models of **(a)** pristine carbon nanotube (CNT), **(b)** effective potential distribution of CNT, **(c)** activated carbon nanotube (ACNT) with surface oxygen-containing functional groups, and **(d)** corresponding effective potential distribution of ACNT.

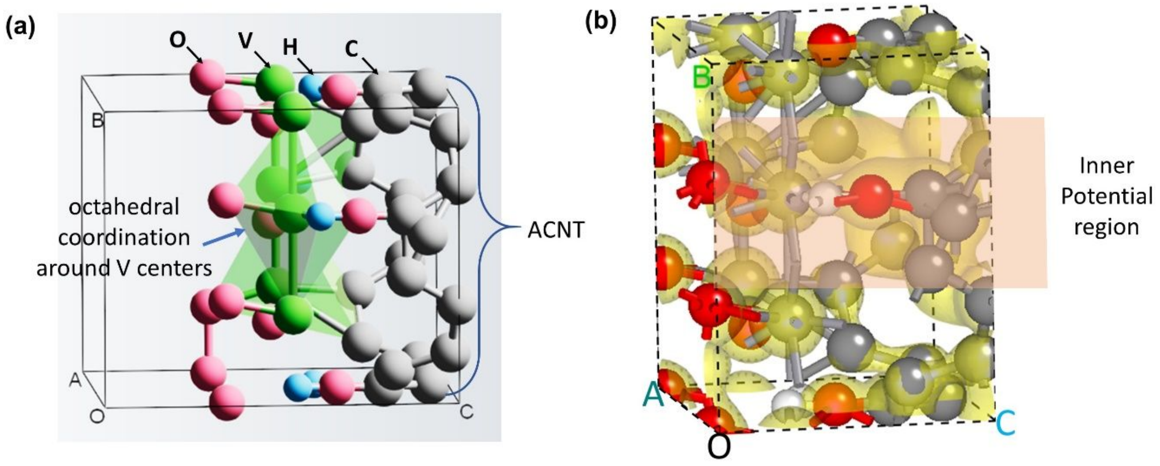

Figure S3. DFT-simulated atomic structure model of **(a)** V_2O_5-ACNT showing octahedral coordination (green polyhedra) representing active catalytic sites, **(b)** effective potential distribution of V_2O_5-ACNT highlighting inner low-potential regions responsible for charge localization and preferential reactant adsorption.

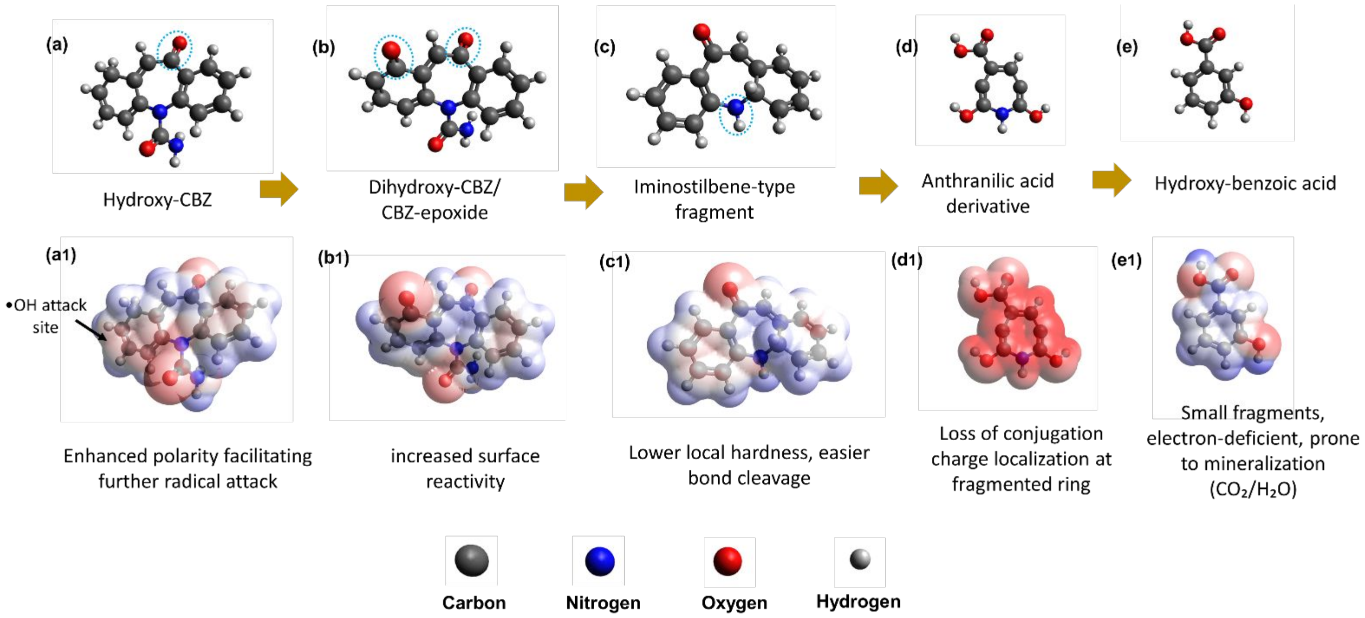

Figure S4. (a-e) DFT-optimized successive removal intermediates of CBZ, **(a1-e1)** Corresponding electrostatic surface potential (ESP) maps highlighting reactive sites for radical attack.

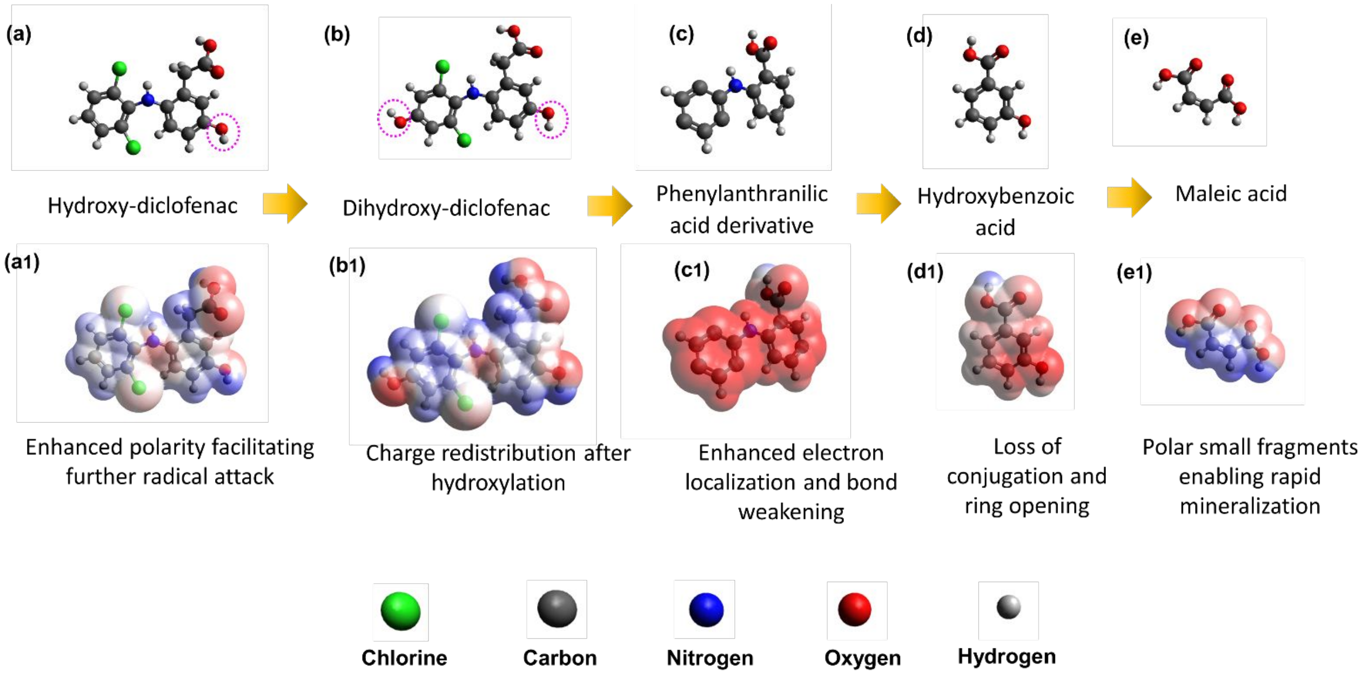

Figure S5. (a) DFT-optimized successive removal intermediates of DFN, **(a1-e1)** Corresponding ESP distributions illustrating charge redistribution and preferential bond-cleavage regions.

(a)

(b)

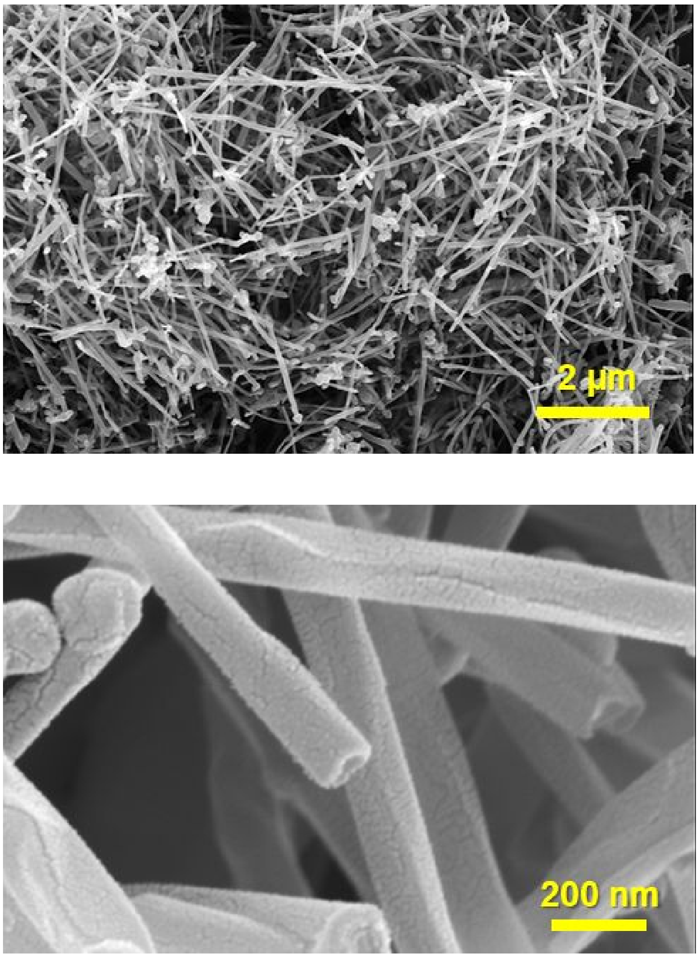

Figure S6. FESEM images of CNTs: **(a)** Entangled CNT network at a magnification of 2 µm, **(b)** High-magnification image at 200 nm, highlighting the tubular morphology and surface texture of individual CNTs.

(a)

(b)

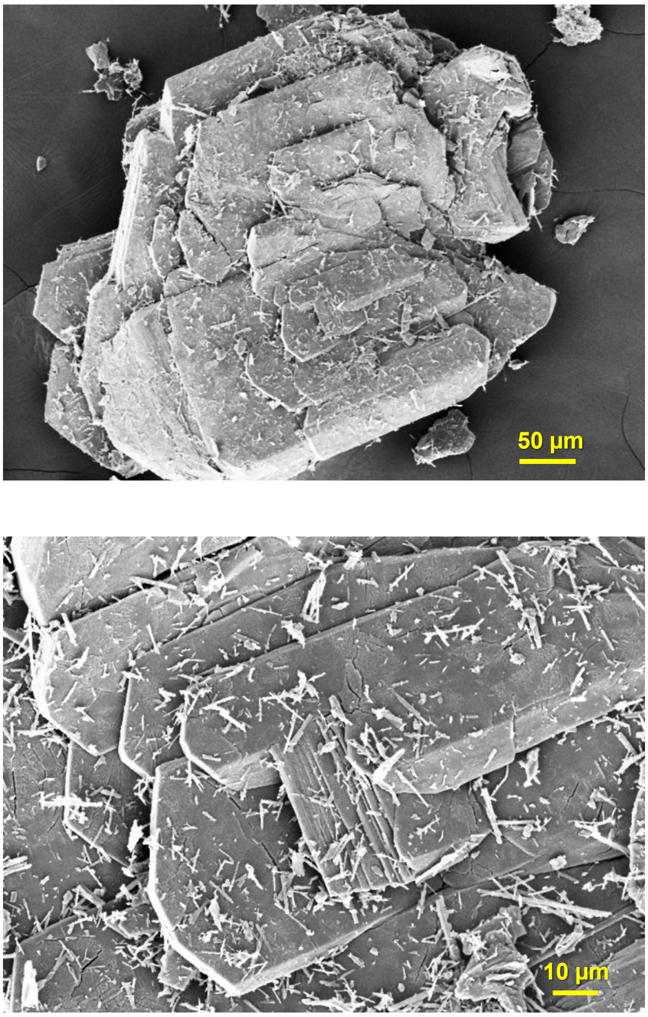

Figure S7. FESEM images of V_2O_5-ACNT1: **(a)** Stacked, plate-like V_2O_5 structures decorated with CNTs, **(b)** High-magnification image revealing the surface morphology and uniform distribution of CNTs on the V_2O_5 plates.

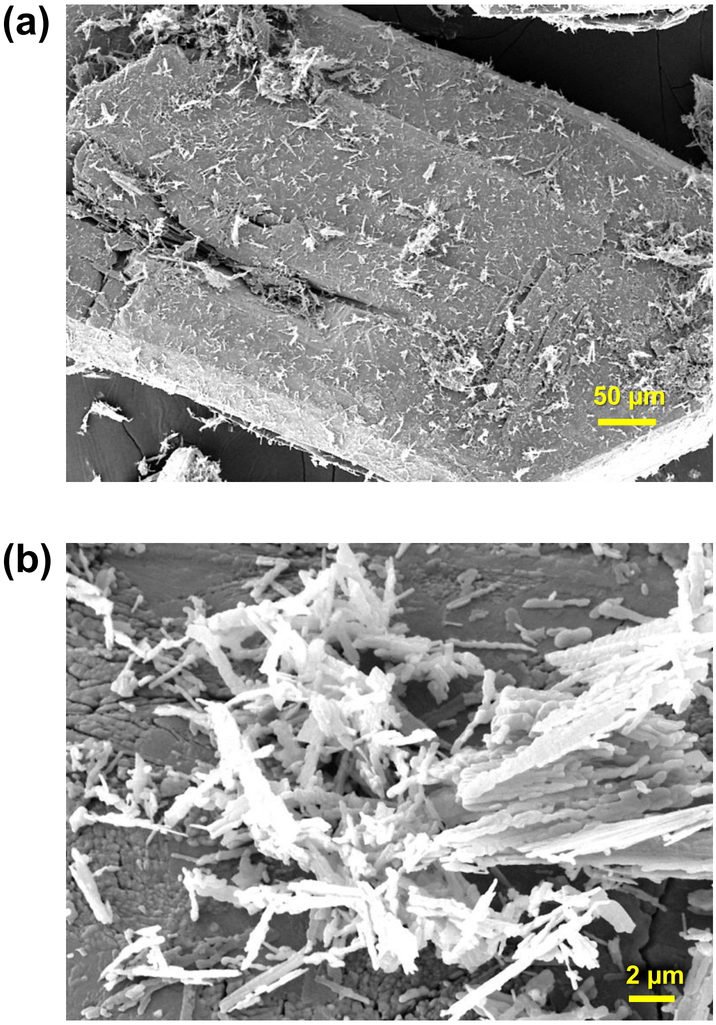

Figure S8. FESEM images of V_2O_5-ACNT5: **(a)** Layered V_2O_5 plate morphology with surface roughening, **(b)** High-magnification image highlighting the dense anchoring and interwoven distribution of CNTs on the V_2O_5 surface.

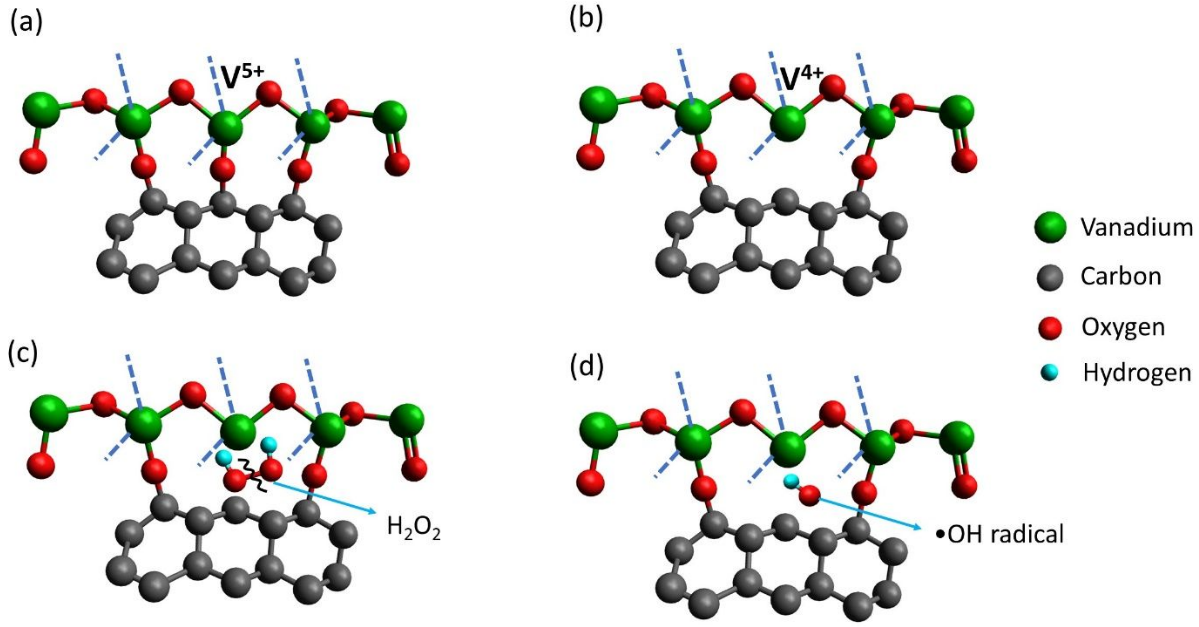

Figure S9. (a) Initial V_2O_5-ACNT interface showing V^{5+} states, **(b)** Creation of an oxygen vacancy resulting in localized V^{4+} sites, **(c)** Interaction of H_2O_2 molecule at the V_2O_5-ACNT interface, **(d)** Generation of a hydroxyl radical (•OH) at the surface.

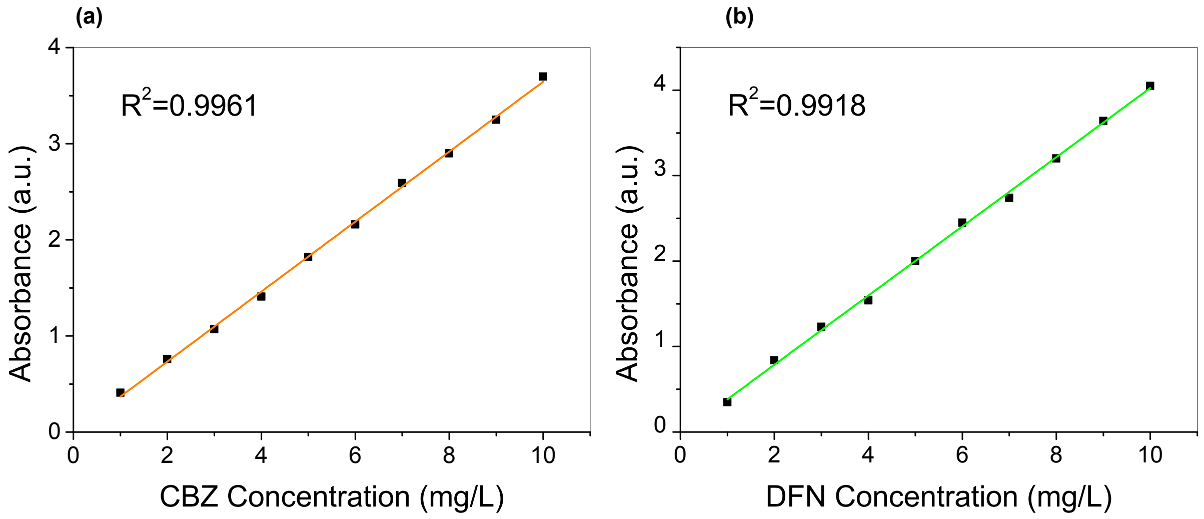

Figure S10. Calibration curves of **(a)** CBZ at 285 nm and **(b)** DFN at 276 nm, showing linear correlation between absorbance and concentration for quantitative analysis.

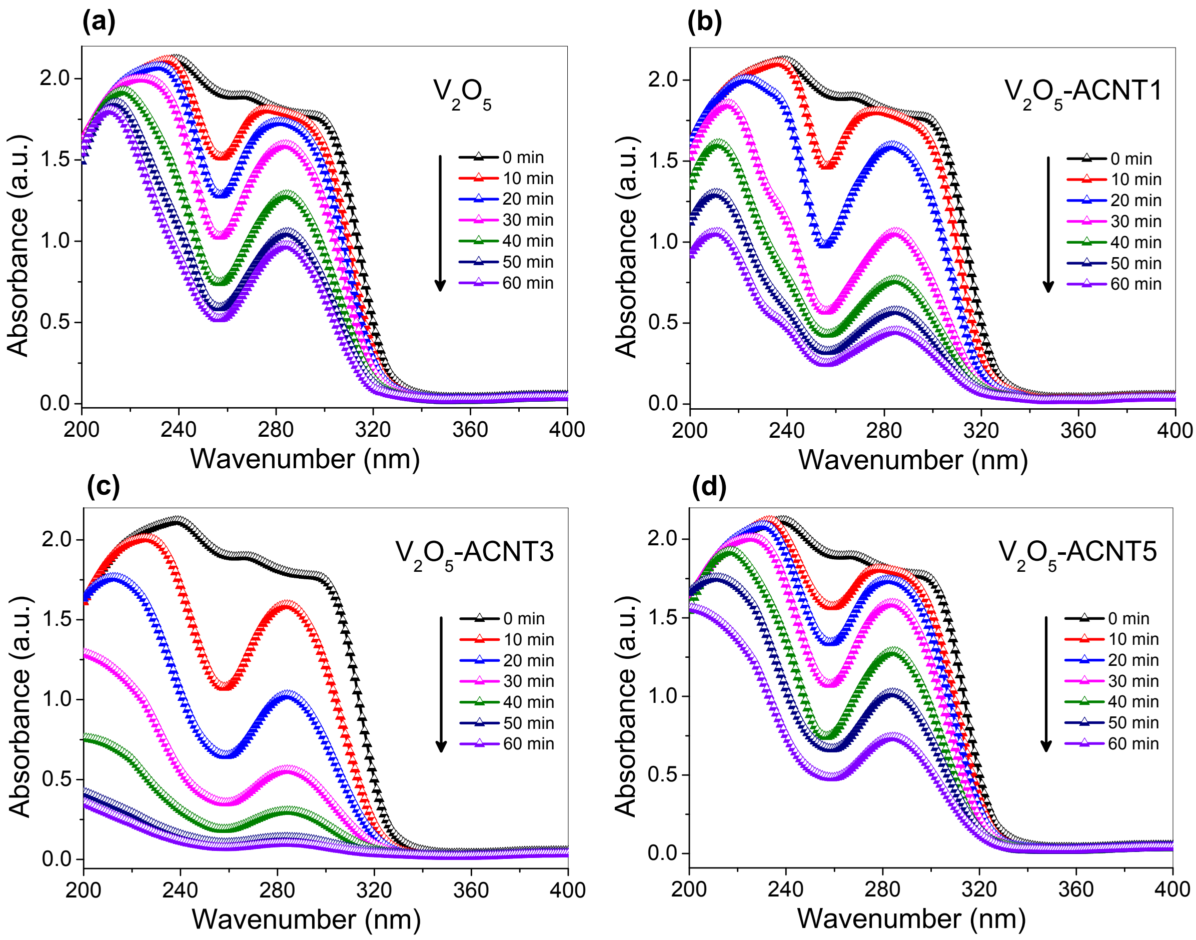

Figure S11. Photocatalytic removal of CBZ under solar light irradiation within 60 min by catalysts, **(a)** V_2O_5, **(b)** V_2O_5-ACNT1, **(c)** V_2O_5-ACNT3, **(d)** V_2O_5-ACNT5.

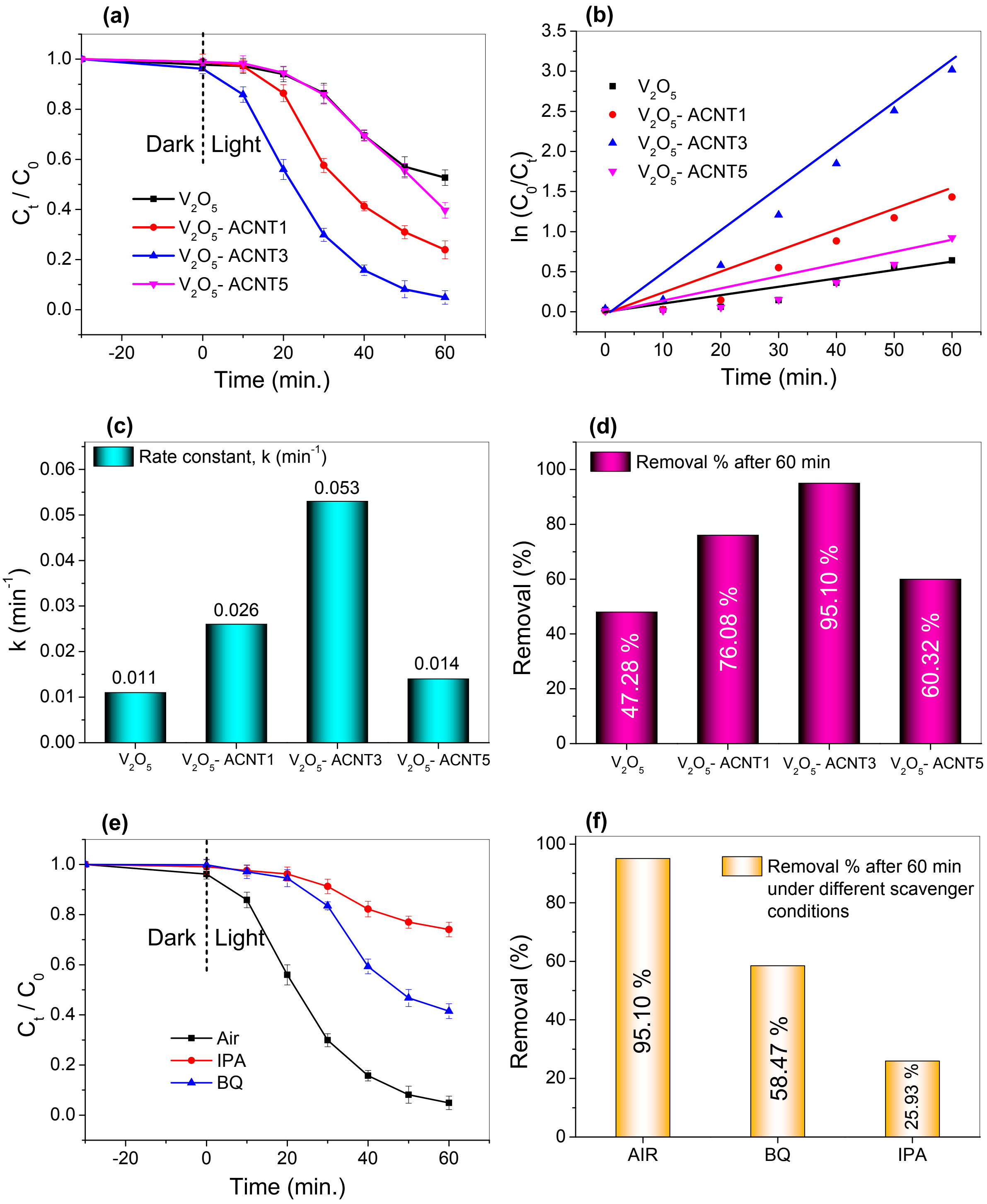

Figure S12. (a) Relative concentration (C_t/C_0) of CBZ with respect to time (min) in light-off/-on states, **(b)** first-order kinetic graphs, *ln*(C_0/C_t) versus time (min), **(c)** removal rate constant demonstrating the highest activity for V_2O_5-ACNT3 against CBZ, **(d)** CBZ removal % by catalysts, **(e-f)** Effect of radical scavengers (BQ and IPA) on CBZ removal efficiency after 60 min, indicating the role of $\bullet O_2^-$ and $\bullet OH$ species.

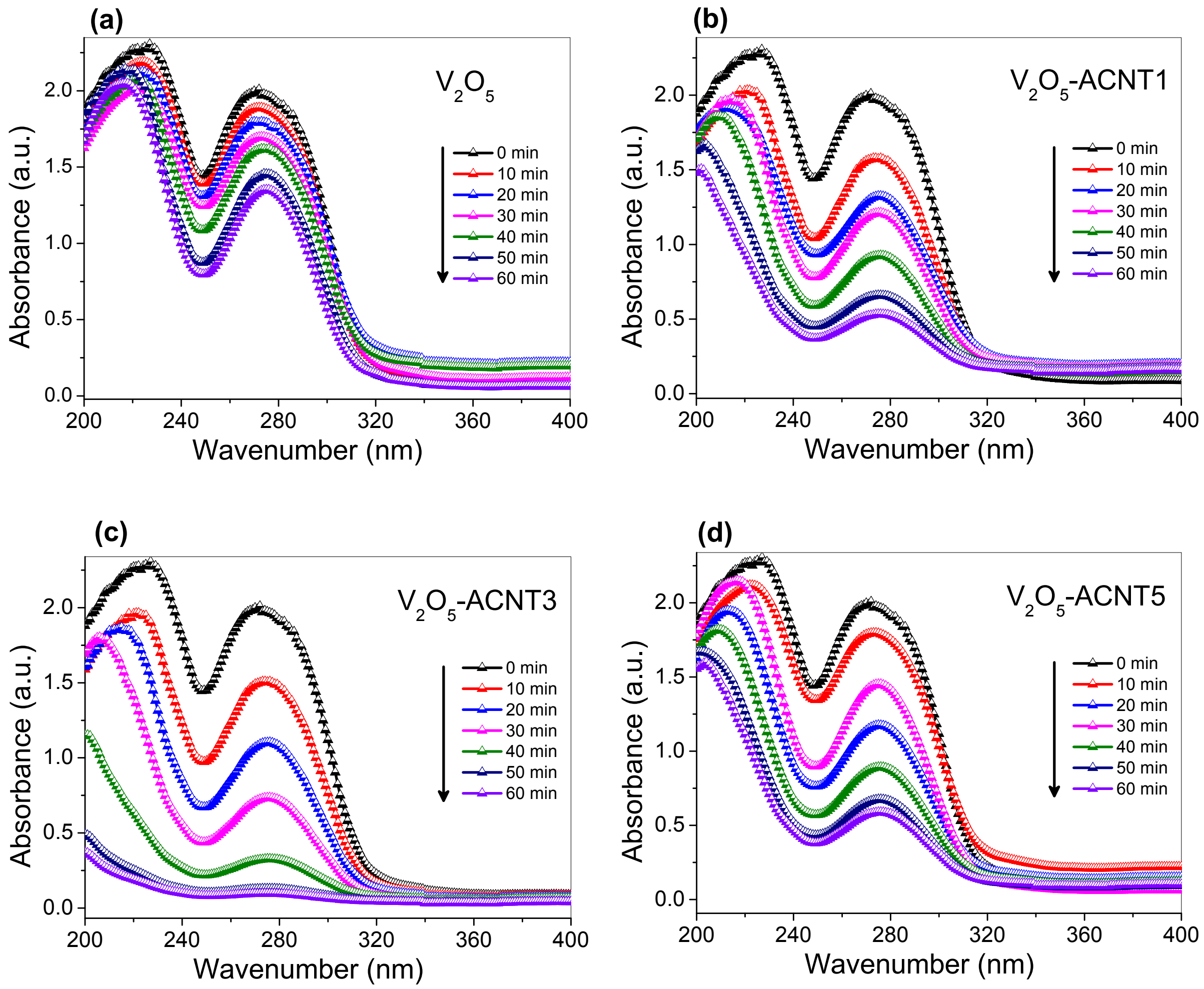

Figure S13. Photocatalytic removal of DFN under solar light irradiation within 60 min by catalysts, **(a)** V_2O_5, **(b)** V_2O_5-ACNT1, **(c)** V_2O_5-ACNT3, **(d)** V_2O_5-ACNT5.

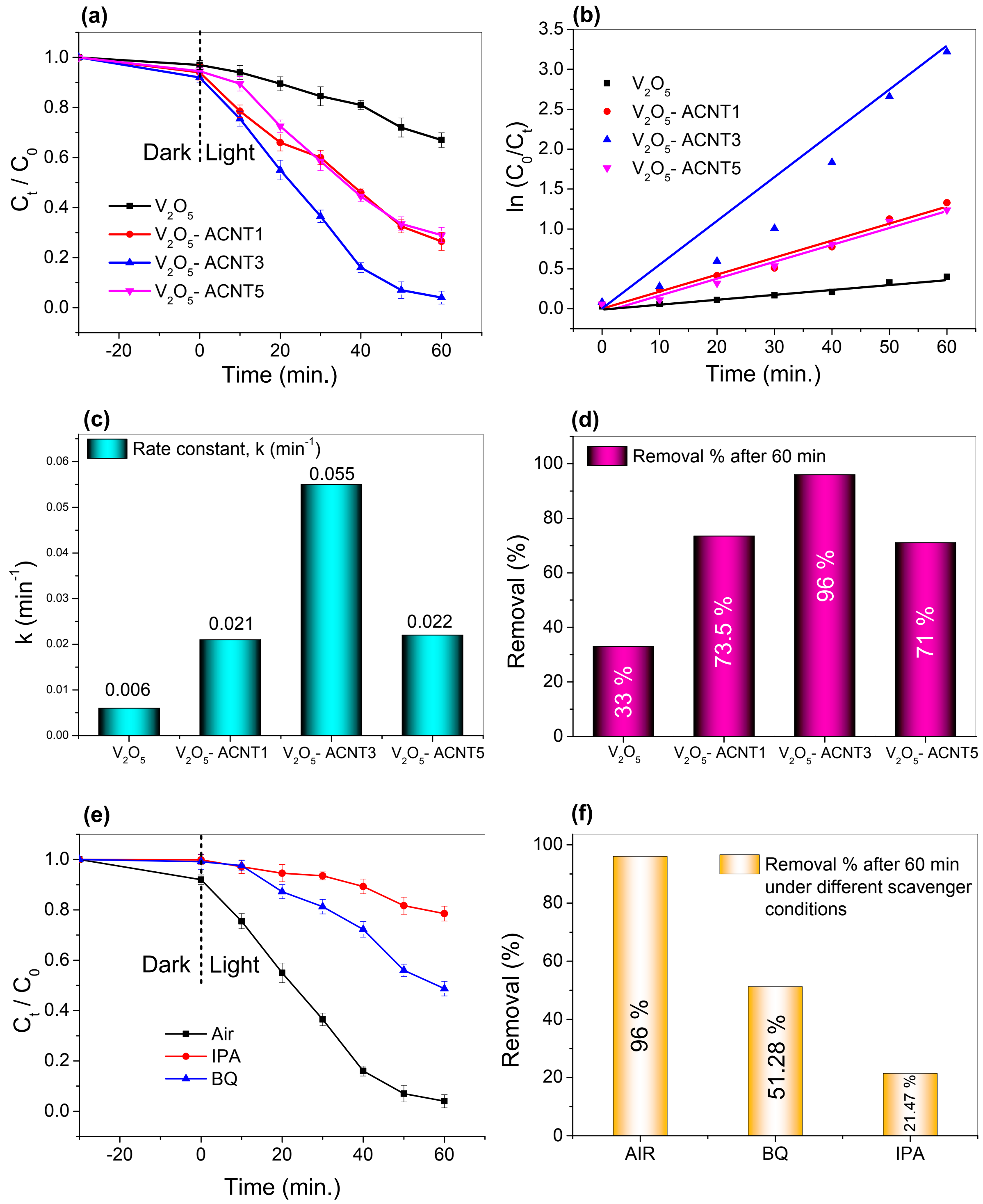

Figure S14. (a) Relative concentration (C_t/C_0) of DFN with respect to time (min) in light-off/-on states, **(b)** first-order kinetic graphs, *ln*(C_0/C_t) versus time (min), **(c)** removal rate constant demonstrating the highest activity for V_2O_5-ACNT3 against DFN, **(d)** DFN removal % by catalysts, **(e-f)** Effect of radical scavengers (BQ and IPA) on DFN removal efficiency after 60 min, indicating the role of $\bullet O_2^-$ and $\bullet OH$ species.

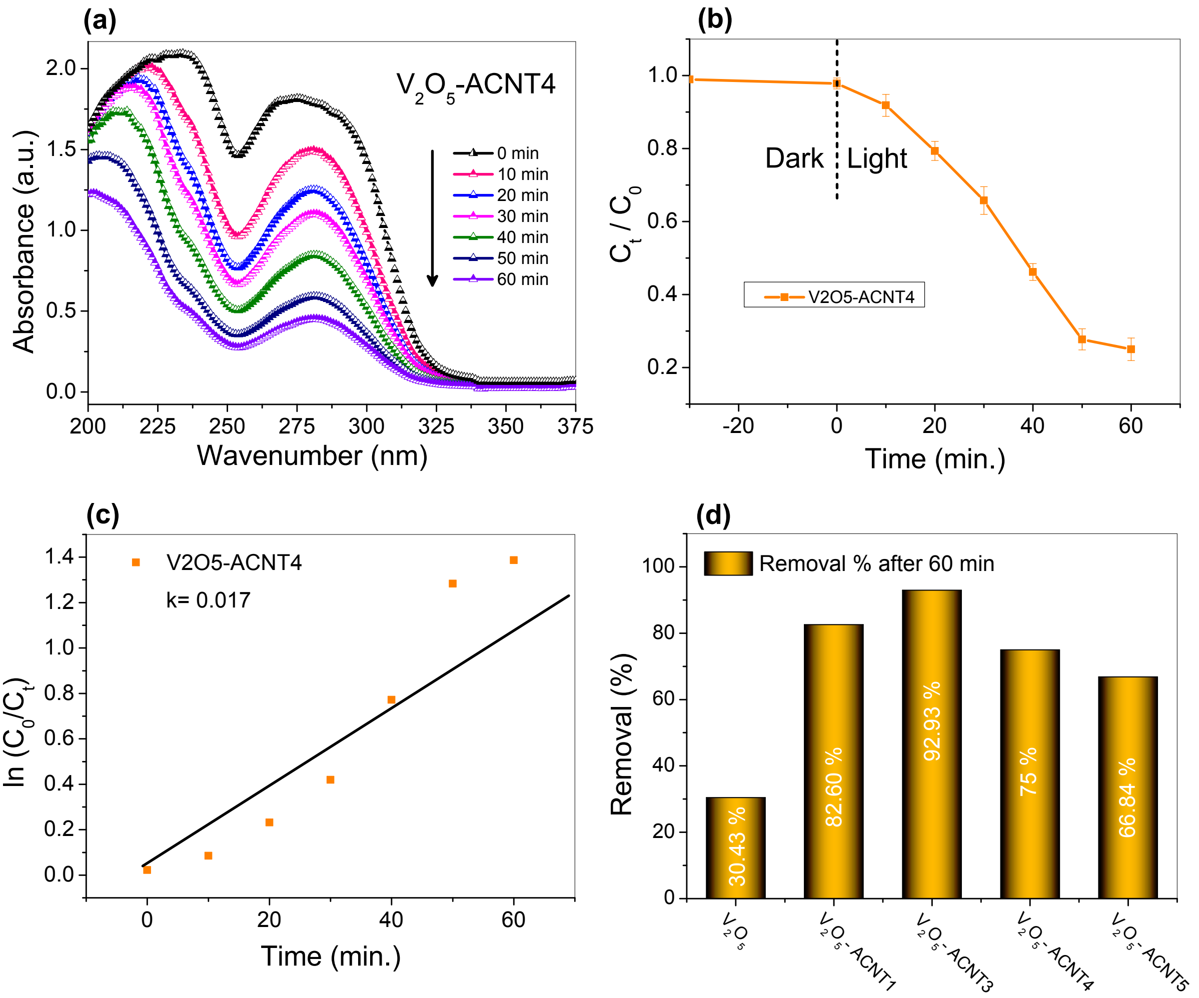

Figure S15. (a) Photocatalytic removal of mixed pollutants (CBZ and DFN) under solar light irradiation within 60 min using V_2O_5-ACNT4 catalyst, **(b)** relative concentration (C_t/C_0) of mixed pollutant as a function of time (min) with catalyst V_2O_5-ACNT4, **(c)** Pseudo-first-order kinetic plots: $\ln(C_0/C_t)$ versus time, **(d)** Photocatalytic removal efficiency (%) of mixed pollutant within 60 min of solar irradiation by V_2O_5-ACNT4.

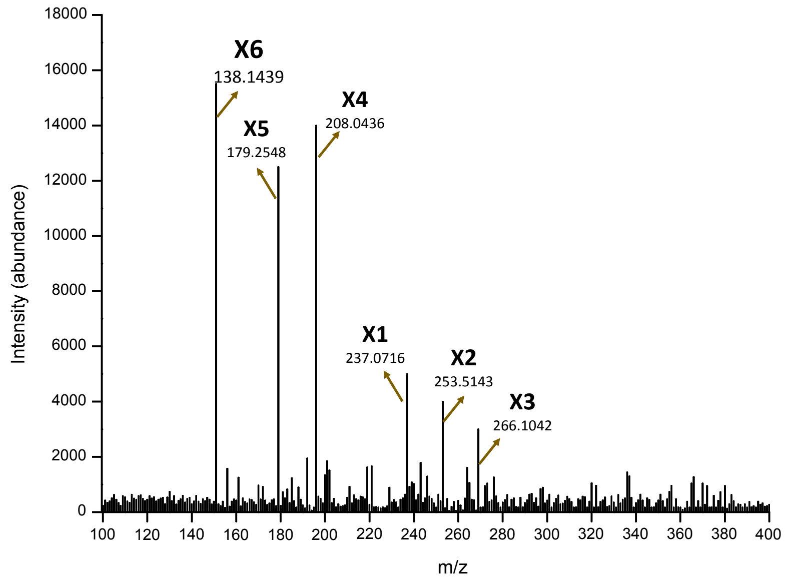

Figure S16. MS results of CBZ intermediate products.

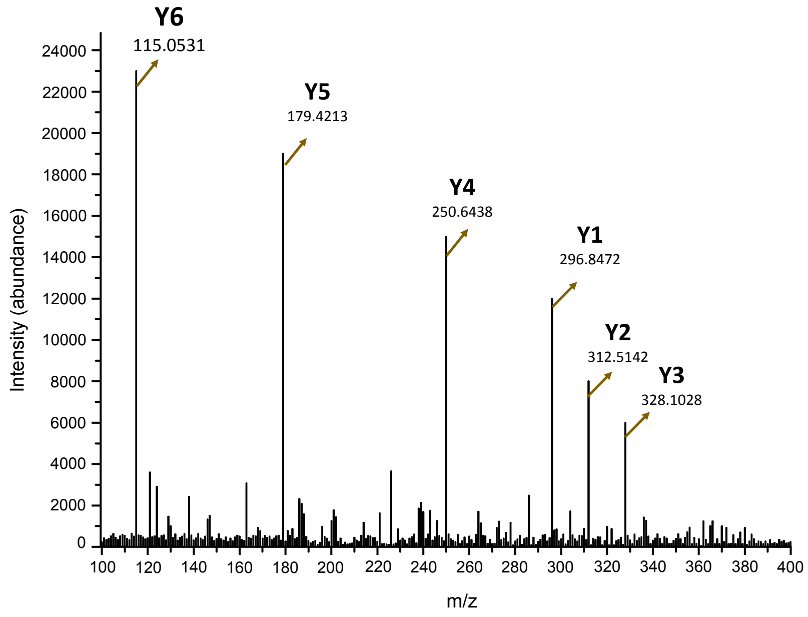

Figure S17. MS results of DFN intermediate products.

Table S1. The values of bandgap (E_g) and Urbach energy (E_u) for prepared samples (ACNT, V_2O_5, V_2O_5-ACNT1, V_2O_5-ACNT3, and V_2O_5-ACNT5).

Samples	Bandgap, E_g [eV]	Slope	Urbach Energy, E_u [eV]
ACNT	0.61	1.46	0.684
V_2O_5	3.71	4.74	0.210
V_2O_5-ACNT1	3.55	2.36	0.423
V_2O_5-ACNT3	2.58	0.80	1.250
V_2O_5-ACNT5	1.98	1.42	0.704

Table S2. Average pore size, surface area, and pore volume of (ACNT, V_2O_5, V_2O_5-ACNT1, V_2O_5-ACNT3, and V_2O_5-ACNT5) catalysts.

Catalyst	Average Pore size [nm]	Surface area [m^2 g^{-1}]	Pore volume [cm^3 g^{-1}]
ACNT	10.27	8.49	20.27
V_2O_5	2.37	1.01	9.46
V_2O_5-ACNT1	6.74	4.11	10.02
V_2O_5-ACNT3	8.64	6.81	16.85
V_2O_5-ACNT5	7.23	5.12	12.31

Table S3. CBZ removal products by mass spectra results.

Compound	m/z	Chemical formula	Intermediate group	Transformation step	Structure
X1	237	$C_{15}H_{12}N_2O$	Carbamazepine (CBZ)	Parent compound	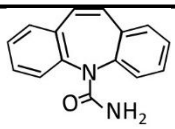
X2	253	$C_{15}H_{12}N_2O_2$	Oxcarbazepine	•OH radical hydroxylation (V^{5+}/V^{4+}mediated)	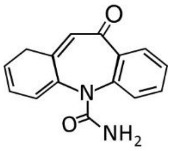
X3	269	$C_{15}H_{10}N_2O_3$	quinone derivative of oxcarbazepine	Successive •OH oxidation	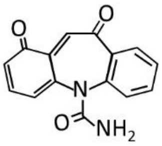
X4	196	$C_{14}H_{11}NO$	Iminostilbene-type fragment	Amide bond cleavage	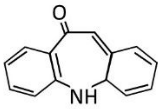
X5	179	$C_7H_7NO_4$	Anthranilic acid derivative	Aromatic ring opening	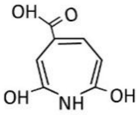
X6	151	$C_7H_6O_3$	Hydroxy-benzoic acid	Further ring fragmentation	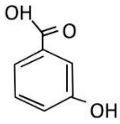

Table S4. DFN removal products by mass spectra results.

Compound	m/z	Chemical formula	Intermediate group	Transformation step	Structure
Y1	296	$C_{14}H_{11}Cl_2NO_2$	Diclofenac (DFN)	Parent compound	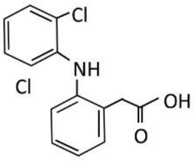
Y2	312	$C_{14}H_{11}Cl_2NO_3$	oxy-diclofenac	•OH hydroxylation	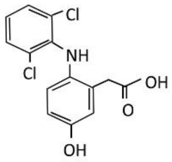
Y3	328	$C_{14}H_{11}Cl_2NO_4$	Dioxy-diclofenac	Successive •OH attack	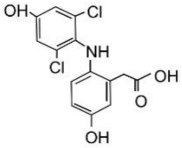
Y4	250	$C_{13}H_{11}NO_2$	Phenylanthranilic acid derivative	C-N bond cleavage	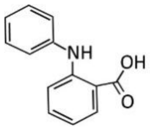
Y5	179	$C_7H_7O_4$	Hydroxybenzoic acid	oxidation	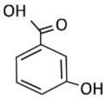
Y6	115	$C_4H_4O_4$	Maleic acid	Aromatic ring cleavage	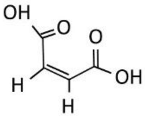

Table S5. Comparison of the catalytic performance of V_2O_5-ACNT Nanohybrids with others catalysts.

**Materials**	**Pollutant**	**Pollutant [mg L⁻¹]**	**Catalyst [mg/10 mL]**	**Light Source**	**Removal Time [min]**	**Removal efficiency [%]**	**Ref.**
g-C_3N_4	Diclofenac	10	20	300 W Xenon arc lamp	60	19.3	1
TiO_2	Diclofenac	50	20	0.7 kW UV lamp	60-80	65	2
CQDs/BiOCOOH	Diclofenac	4	6	350 W Xenon lamp	60	82.4	3
Zn1-x-yPrxAlyO	Diclofenac	30	5	125 W, UV lamp	150	68.14	4
Mn-WO_3	Diclofenac	2	2.2	LED Visible Light	180	88	5
TiO_2 /g-C_3N_4	Diclofenac	5	-	15 W, mercury lamp	90	93.49	6
TiO_2-CdS	Diclofenac	-	-	15 W, mercury lamp	240	86	7
CuS/NiS	Diclofenac	15	20	15 W, mercury lamp	120	68.45	8
AgI/CeO_2	Diclofenac	-	-	125 W, UV light	120	97	9
TiO_2-SnO_2	Diclofenac	20	8	125 W, UV light	300	90	10
$Ce_{0.93}Sb_{0.07}FeO_3$	Diclofenac	5	15	15 W, mercury lamp	120	86.7	11
M-SiO_2/g-C_3N_4	Carbamazepine	-	10	300 W, Xenon lamp	60	78.92	12
g-C_3N_4/TiO_2	Carbamazepine	-	-	125 W, UV light	360	71.41	13
Pec/CS/ZnO	Carbamazepine	10	5	150 W, Halogen lamp	180	69.5	14
MIL-68-NH_2/Bi_4O_7	Carbamazepine	50	10	500 W, Xenon lamp	120	92.7	15
g-C_3N_4/$Bi_2O_2CO_3$	Carbamazepine	20	40	150 W, Halogen lamp	180	98	16
FeS_2/Fe_2O_3	Carbamazepine	2.5	8	300 W Xenon lamp	60	65	17
Ce-doped ZnO	Carbamazepine	-	-	15 W, mercury lamp	180	53	18
**V_2O_5-ACNT3**	**Carbamazepine**	**50**	**5**	**150 W Halogen lamp**	**60**	**95.10**	**This Work**
**V_2O_5-ACNT3**	**Diclofenac**	**50**	**5**	**150 W Halogen lamp**	**60**	**96**	**This Work**

References

(1) Liu, W.; Li, Y.; Liu, F.; Jiang, W.; Zhang, D.; Liang, J. Visible-light-driven photocatalytic degradation of diclofenac by carbon quantum dots modified porous g-C3N4: Mechanisms, degradation pathway and DFT calculation. *Water Res.* **2019**, *151*, 8-19.
(2) Czech, B.; Rubinowska, K. TiO2-assisted photocatalytic degradation of diclofenac, metoprolol, estrone and chloramphenicol as endocrine disruptors in water. *Adsorpt.* **2013**, *19* (2), 619-630.
(3) Chen, P.; Zhang, Q.; Su, Y.; Shen, L.; Wang, F.; Liu, H.; Liu, Y.; Cai, Z.; Lv, W.; Liu, G. Accelerated photocatalytic degradation of diclofenac by a novel CQDs/BiOCOOH hybrid material under visible-light irradiation: Dechloridation, detoxicity, and a new superoxide radical model study. *Chem. Eng. J.* **2018**, *332*, 737-748.
(4) Martins, E.; Trigueiro, P.; Jerônimo, A. G.; Barbosa, R.; Neves, L.; Sales, D. A.; Almeida, L. C.; Viana, B. C.; Soares, A. S.; Peña-Garcia, R. R. Efficient photocatalytic degradation of diclofenac drug using the Zn1-x-yPrxAlyO photocatalyst under UV light irradiation. *Environ. Sci. Pollut. Res.* **2024**, *31* (40), 53074-53089.
(5) Yazdanbakhsh, A.; Eslami, A.; Massoudinejad, M.; Gholami, Z.; Sarafraz, M.; Noorimotlagh, Z.; Adiban, M.; Mirzaee, S. A. Photocatalytic degradation and dechlorination mechanism of diclofenac using heterojunction Mn-doped tungsten trioxide (Mn-WO3) nanoparticles under LED visible light from aqueous solutions. *Sci. Rep.* **2024**, *14* (1), 29583.
(6) John, P.; Johari, K.; Gnanasundaram, N.; Appusamy, A.; Thanabalan, M. Enhanced photocatalytic performance of visible light driven TiO2/g-C3N4 for degradation of diclofenac in aqueous solution. *Environ. Technol. Innov.* **2021**, *22*, 101412.
(7) Elangovan, M.; Bharathaiyengar, S. M.; PonnanEttiyappan, J. Photocatalytic degradation of diclofenac using TiO2-CdS heterojunction catalysts under visible light irradiation. *Environ. Sci. Pollut. Res.* **2021**, *28* (14), 18186-18200.
(8) Farahani, B.; Giahi, M.; Ghorbani, M.; Fazaeli, R.; Moradi, O. Synthesis of CuS/NiS heterostructural photocatalyst and its performance in the degradation of metronidazole and diclofenac drugs: optimization of operating conditions. *J. Nanostruct. Chem.* **2023**, *13* (2), 303-320.
(9) Chaudhari, S. M.; Gonsalves, O. S.; Nemade, P. R. Enhanced photocatalytic degradation of Diclofenac with AgI/CeO2: a comparison with Mn, Cu and Ag-doped CeO2. *Mater. Res. Bull.* **2021**, *143*, 111463.
(10) Mugunthan, E.; Saidutta, M.; Jagadeeshbabu, P. Photocatalytic degradation of diclofenac using TiO2–SnO2 mixed oxide catalysts. *Environ. Technol.* **2019**, *40* (7), 929-941.
(11) Javaid, A.; Imran, M.; Kanwal, F.; Latif, S. Antimony-doped cerium ferrite: a robust photocatalyst for the mitigation of diclofenac potassium, an emerging contaminant. *Mater. Sci. Semicond. Process.* **2024**, *177*, 108350.
(12) Li, R.; Wu, Z.; Yang, Y.; Sun, S.; Ma, R.; Ding, H. Photocatalytic persulfate activation by silica microsphere-supported g-C3N4 for efficient carbamazepine degradation. *Mater. Sci. Semicond. Process.* **2024**, *184*, 108792.
(13) Kane, A.; Chafiq, L.; Dalhatou, S.; Bonnet, P.; Nasr, M.; Gaillard, N.; Dikdim, J. M. D.; Monier, G.; Assadi, A. A.; Zeghioud, H. g-C3N4/TiO2 S-scheme heterojunction photocatalyst with enhanced photocatalytic Carbamazepine degradation and mineralization. *J. Photochem. Photobiol. A-Chem.* **2022**, *430*, 113971.
(14) Sandhya, G.; Suresh, D.; Rao, A. M.; Sribalan, R. Chitosan–Schiff Base Cu (II) Complex-Grafted ZnO Nanoparticles as a Photocatalyst for Degrading Pharmaceutical Pollutants Under Diverse Light Sources. *Top. Catal.* **2025**, 1-18.
(15) Zhao, W.; Yan, M.; Chen, Y.; Shen, J.; Hong, X.; Mu, F.; Li, S.; Zhang, S.; Wang, Q.; Dai, B.; et al. Rational design of novel metal-organic framework/Bi4O7 S-scheme heterojunction photocatalyst for boosting carbamazepine degradation. *Appl. Surf. Sci.* **2023**, *622*, 156876.
(16) Abbasi, J. U. R.; Leong, K. H.; Sim, L. C.; Dai, C.; Aziz, A. A.; Sethupathi, S.; Ibrahim, S. Amalgamation of g-C3N4/Bi2O2CO3 heterojunction composites for enhanced sunlight photocatalytic removal of carbamazepine. *Environ. Sci. Pollut. Res.* **2025**, 1-14.

(17) Guo, Q.; Tang, G.; Zhu, W.; Luo, Y.; Gao, X. In situ construction of Z-scheme FeS2/Fe2O3 photocatalyst via structural transformation of pyrite for photocatalytic degradation of carbamazepine and the synergistic reduction of Cr (VI). *J. Environ. Sci.* **2021**, *101*, 351-360.
(18) Caregnato, P.; Jiménez, K. R. E.; Villabrille, P. I. Ce-doped ZnO as photocatalyst for carbamazepine degradation. *Catal. Today* **2021**, *372*, 183-190.
